# Dysregulated transcriptional networks in *KMT2A-* and *MLLT10*-rearranged T-ALL

**DOI:** 10.1186/s40364-018-0141-z

**Published:** 2018-08-23

**Authors:** Huining Kang, Nitesh D. Sharma, Christian K. Nickl, Meenakshi Devidas, Mignon L. Loh, Stephen P. Hunger, Kimberly P. Dunsmore, Stuart S. Winter, Ksenia Matlawska-Wasowska

**Affiliations:** 1 Department of Internal Medicine, University of New Mexico, Albuquerque, NM USA; 2 Department of Pediatrics, University of New Mexico, MSC105590, Albuquerque, NM 87131 USA; 3 Department of Biostatistics, University of Florida, Gainesville, FL USA; 4 Department of Pediatrics, University of California at San Francisco, San Francisco, CA USA; 5 Children’s Hospital of Philadelphia, University of Pennsylvania, Philadelphia, PA USA; 6 Pediatric Hematology/Oncology, University of Virginia, Charlottesville, VA USA; 7 Children’s Minnesota Research Institute, Children’s Minnesota, Minneapolis, MN USA

**Keywords:** Gene expression, KMT2A, MLLT10, Leukemia, Microarray, T-ALL

## Abstract

For children and young adults with T-lineage acute lymphoblastic leukemia (T-ALL), event free survival following relapse is < 10%. We recently showed that rearrangements of the mixed lineage leukemia gene (*KMT2A*-R) are associated with induction failure and an inferior survival in T-ALL. Because there are currently no molecular features that inform treatment strategies in T-ALL, we hypothesized that transcriptional alterations related to *KMT2A-R* and *MLLT10-R* T-ALL could identify biologically relevant genes and signaling pathways for the development of targeted therapies for these groups of patients. We analyzed microarray data from a retrospective cohort of 100 T-ALL patients to identify novel targets for *KMT2A* (*n* = 12) or *MLLT10* (*n* = 9) chimeras. We identified 330 probe sets that could discriminate between these groups, including novel targets, like *RUNX2, TCF4* or *MYO6*. The results were further validated in two independent data sets and the functional networks were analyzed to identify pathways that may be of pathogenic or therapeutic relevance.

**Electronic supplementary material:**

The online version of this article (10.1186/s40364-018-0141-z) contains supplementary material, which is available to authorized users.

## Introduction

Despite our growing understanding of the heterogeneity of genomic alterations in T-ALL [1–5], genetic alterations are not used to stratify therapy in T-ALL. Although, approximately 80-90% of patients with T-ALL can be cured, for those who relapse, event free survival is poor [6]. Because attempts to further dose-intensify therapy have generally resulted in greater toxicity without improved survival, efforts are underway to identify more effective treatments for patients with high-risk disease, including those with high levels of post-induction minimal residual disease [6]. A better understanding of the molecular drivers of resistant disease may inform the development of targeted therapies to improve outcome and reduce the burden of treatment-related acute and chronic adverse events.

The repertoire of chromosomal rearrangements affecting the *KMT2A* gene (*KMT2A*-R) includes over 100 translocation partners [7]. We recently showed that *KMT2A-MLLT4* and del3’*KMT2A* are important determinants of high-risk disease in *HOXA-*deregulated T-ALL [8]. The clinical outcome of other *HOXA*-related lesions involving *MLLT10* gene (*MLLT10-*R), remains controversial [8–10]. Using gene expression profiles, others have developed signatures that distinguish *KMT2A*-R in AML and B-ALL [11, 12] however such studies have not been performed on larger data sets for T-ALL. The data on transcriptional signatures in T-ALL with *KMT2A*-R and *MLLT10*-R is very limited [2–5]. Specifically, gene expression signatures in *KMT2A*-R T-ALL were reported for only three *KMT2A*-*MLLT1* cases thus far [2, 5]. While deregulation of *HOXA9/10* is a hallmark of *KMT2A-R* and *MLLT10-R*, its over-expression does not inform patient outcome, suggesting that additional genes deregulated by these translocations could play an important role in leukemia pathobiology. Therefore, we hypothesized that supervised profiling of 100 well-characterized T-ALL cases could identify *KMT2A-* and *MLLT10*-deregulated genes and signaling networks allowing the development of targeted therapies in T-ALL.

## Material and methods

Primary T-ALL samples were obtained from patients enrolled in Children’s Oncology Group AALL0434 study (*n* = 100) [8]. All cases that passed the hybridization quality controls were subjected to Affymetrix U133 Plus 2.0 microarray [13]. Scale factor < 40; *GAPDH* M33197 3′ intensity > 15,000; and *GAPDH* M33197 3′/5′ ratio < 3 were applied as array experimental quality parameters [13]. The Robust Multi-array Average (RMA) algorithm was used to generate and normalize signal intensities [8, 13]. From 54,675 probe sets, we selectively filtered out probes associated with gender-related genes, globins, and internal controls [8, 13]. Linear Models for the Microarray approach implemented in R package limma [14] was utilized to identify differentially expressed probe sets in association to specific genomic lesions. Benjamini and Hochberg method was used to calculate the False Discovery Rate (FDR) to adjust for multiple testing. Java Treeview [15] was used to generate the heatmaps. Gene Set Enrichment Analysis (GSEA) [16] was performed to identify signaling pathways related to specific genomic lesions.

## Results and discussion

**Fig. 1:**
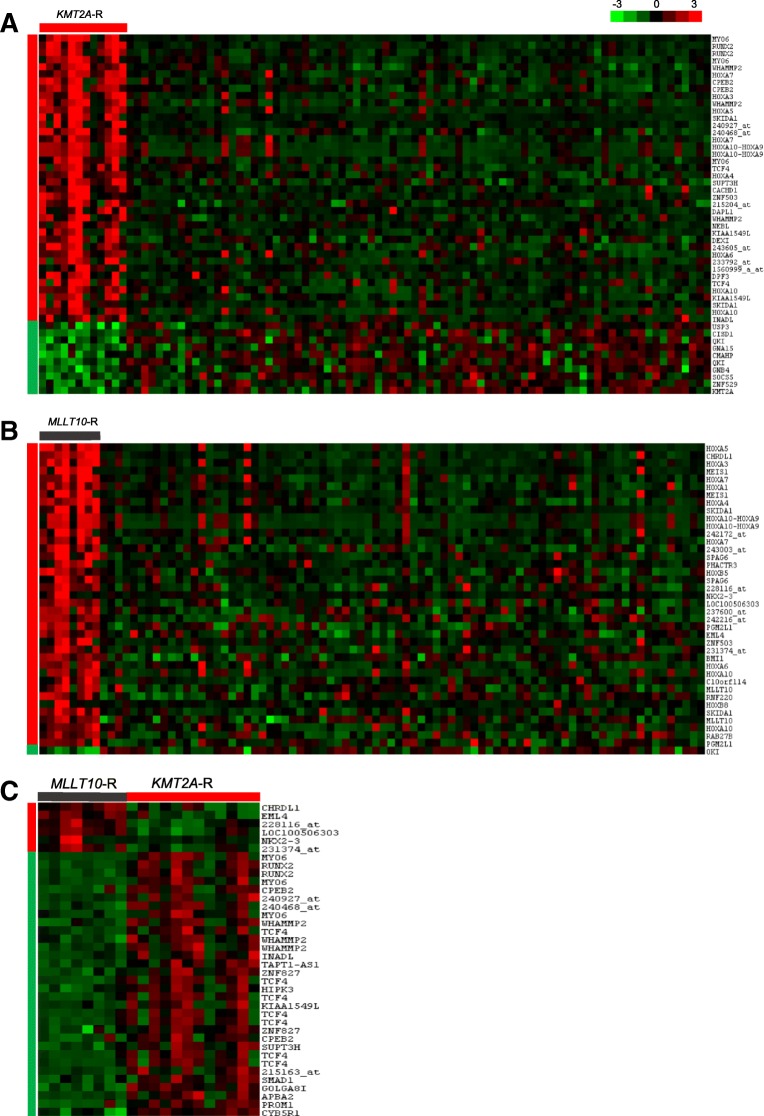
Gene expression profiling for *KMT2A*-R and *MLLT10*-R in a cohort of 100 T-ALL samples. Analyses were performed for 330 probe sets related to commonly and exclusively differentially expressed genes in 3 T-ALL groups: *KMT2A*-R, *MLLT10*-R and Others (FDR ≤ 0.05) (Additional file 2: Table S1). **a** Heat map of the top 40 up-regulated and top 10 down-regulated genes in *KMT2A*-R as compared to cases that do not harbor either *KMT2A*- or *MLLT10*-R. The genes were selected out of 307 identified probe sets ranked by FDR ≤0.05 **b** Heat map indicating 46 probe sets associated with aberrantly deregulated genes in *MLLT10*-R as compared to cases not harboring these and *KMT2A* gene lesions. **c** Differentially expressed genes (27 probe sets) in *MLLT10*-R versus *KMT2A*-R. Columns indicate T-ALL samples organized in groups based on presence or absence of genomic lesion of interest. “Others” reflect cases that do not have either *KMT2A*-R or *MLLT10*-R. Rows indicate probe sets corresponding to significantly expressed genes. Each row represents the relative expression for a particular gene across the samples within above the mean (red), below the mean (green), and around the mean (black). Vertical bars discriminate between up-regulated (red) and down-regulated (green) genes in given comparable groups

Because *KMT2A-R* and *MLLT10-R* drive *HOXA*-deregulated leukemias, we sought to identify specific genes that are enriched in T-ALL with these genomic abnormalities. Specifically, we searched for differentially expressed genes that could discriminate between T-ALL cases with *KMT2A-*R (*n* = 12), *MLLT10-*R (*n* = 9) and the remaining T-ALL cases lacking these alterations (Others; *n* = 79), and found 330 probe sets corresponding to genes deregulated between these groups (False Discovery Rate; FDR ≤0.05) (Additional file 1: Figure S1A, Additional file 2: Table S1). For T-ALL samples harboring *KMT2A*-R, 258 probes sets were found significantly differentially expressed including 242 probe sets that were upregulated and only 16 probe sets corresponding to genes downregulated in *KMT2A*-R (Fig. 1a). In addition to *HOXA* genes, *KMT2A*-R had increased expression of *PROM1*, encoding transmembrane glycoprotein, *MYO6,* which encodes ATP-dependent motor protein, and multiple regulators of transcription: *RUNX2, TCF4, ZNF503, ZNF827, SMAD1, CPEB2.* We also identified increased expression of *WHAMMP2/WHAMMP3* and *GOLGA8I* pseudogenes located on chromosome 15q13.1, which were recently reported in AML [7]. Thirty-nine probe sets were upregulated and only one was downregulated in *MLLT10*-R cases (Fig. 1b). In agreement with others, *MLLT10*-R differentially expressed *HOXA* genes, *MEIS1,* and other genes located at chromosome 10: *CASC10*, *SKIDA1, SPAG6, ZNF503, BMI1 and COMMD3* [4]. *QKI*, which encodes an RNA-binding protein involved in alternative splicing, was found to be the most downregulated gene in both *KMT2A-*R and *MLLT10-*R cases (Fig. 1a, b). All the above genes except for *BMI1, COMMD3*, *NKX2.3* and *EML* were also deregulated in *KMT2A*-R cases indicating that *KMT2A-R and MLLT10-R* share similar transcription programs when compared to lesion-negative cases.

Because *KMT2A*-R and *MLLT10*-R demonstrate a strong similarity in *HOXA*-mediated deregulation of gene expression, we compared gene expression profiles between these two groups. Among 38 probe sets shown in Fig. 1c, thirty-two were downregulated and five were upregulated in *MLLT10-*R compared to *KMT2A*-R. *MYO6, RUNX2, CPEB2, ZNF827* and *TCF4* were the most overexpressed genes in *KMT2A-*R compared to *MLLT10*-R, suggesting that *KMT2A*-R T-ALL encompass a specific biological subset, which collectively drive a unique oncogenic program. Since *KMT2A-MLLT4* confers an inferior outcome, we sought to determine which genes discriminate between *KMT2A-MLLT4* (*n* = 5) and *KMT2A-MLLT1* (n = 5). Among the 26 discriminatory probes sets, we found two, *MLLT4* and uncharacterized *RP11-38P22* that were over-expressed and 24 were downregulated, including *SEPW1, SMAD1, CHI3L2* and *MYOM2* between *MLLT4* and *MLLT1* (Additional file 1: Figure S1B).

To validate our findings in an independent patient cohort we have performed differential gene expression profiling using existing T-ALL microarray data sets reported by Soulier [2] and Dik [3]. The Soulie’s data set consisted of 92 T-ALL cases including 3 harboring *KMT2A*-*MLLT1* and four with *PICALM-MLLT10* alterations. The Dik’s data set comprised of 23 cases including six *PICALM-MLLT10* fusions*.* We observed a significant overlap between our data and existing microarray data sets confirming that in T-ALL, KMT2A and MLLT10 chimeras drive unique transcriptional programs resulting in specific changes in gene signatures and expression patterns (Additional file 3: Table S2, Additional file 4: Table S3).

**Fig. 2:**
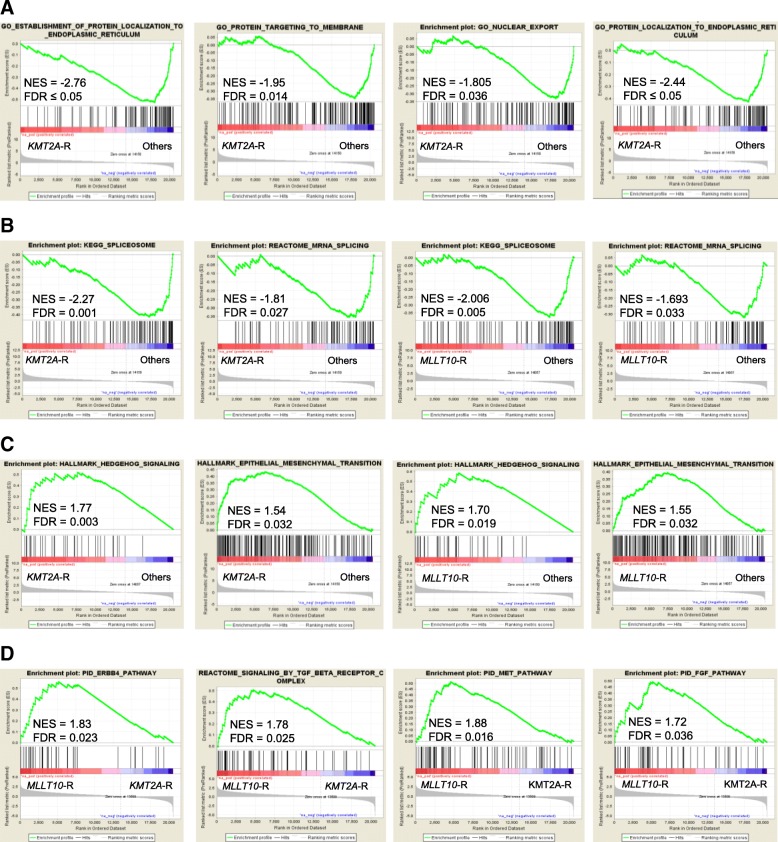
*KMT2A*-R and *MLLT10*-R T-ALL show dysregulation in multiple cell signaling pathways critical for leukemia development and progression. **a** Negatively correlated gene sets in *KMT2A*-R T-ALL (intracellular transport). Green line shows the enrichment score based on hits of genes (indicated by the bars on the abscissa) in the ordered list of differentially regulated genes resulting from the comparison of *KMT2A-*R positive samples and Other T-ALL patient samples. Red/blue bar area beneath the black bars indicates degree of association with a specific phenotype. **b** *KMT2A*-R and *MLLT10*-R are negatively enriched in genes involved in alternative splicing compared to the Others. **c** Example of positively enriched gene sets containing genes upregulated in both *KMT2A*-R and *MLLT10*-R samples compared to the Others. **d** Positively correlated gene sets in *MLLT10*-R vs. *KMT2A*-R. Gene sets containing genes upregulated in *MLLT10*-R positive samples compared to *KMT2A*-R samples (distinct tyrosine kinase signaling pathways). NES, Normalized Enrichement Score; FDR, False Discovery Rate

To further characterize transcriptional alterations in *MLLT10*-R and *KMT2A*-R T-ALL, we performed GSEA to assess functional networks and aberrant cell signaling pathways [16]. Gene ontology and canonical pathway analyses identified multiple genes and signaling networks, which were commonly or exclusively dysregulated in *KMT2A*-R and/or *MLLT10*-R cases. *KMT2A*-R were negatively enriched in regulators of protein export, intracellular protein localization and transport (Fig. 2a). Aberrant localization of oncoproteins or tumor suppressors have been detected in many different types of cancer [17]. Thus, downregulation in protein transport machinery may lead to the disruption in signal transduction in *KMT2A*-R T-ALL. On the contrary, genes upregulated in *KMT2A-*R included regulators of extracellular matrix organization and collagen formation, which are known modulators of cancer invasion (Additional file 5: Table S4). The tumor microenvironment and adhesion were also shown to play a protective role in conferring drug resistance in leukemia [18].

Interestingly, *MLLT10-*R were positively enriched in regulators of embryonic development, while genes specifically downregulated in this group were mostly associated with cell cycle, DNA synthesis and repair (Additional file 5: Table S4, Additional file 6: Table S5) [4]. Genes involved in protein K48-linked ubiquitination and stem cell differentiation were positively enriched in *MLLT10-*R when compared to *KMT2A*-R (Additional file 7: Figure S2). Both, *MLLT10*-R and *KMT2A-*R showed downregulation in a number of genes involved in the regulation of gene expression, ribosome organization and biogenesis, and chromatin modifications (Additional file 5: Table S4, Additional file 6: Table S5). Importantly, we found that *KMT2A*-R and *MLLT10*-R were negatively enriched in genes involved in alternative splicing and mRNA processing (Fig. 2b). These findings might be linked to the downregulation of the *QKI* gene as seen in lung and brain tumors [19]. Deregulation of alternative splicing promotes genomic instability leading to the generation of aberrantly spliced genes and subsequent malignant transformation [20].

Recently, a subset of T-ALL cases with the activation in the hedgehog pathway was shown to be sensitive to the hedgehog pathway inhibitor, vismodegib [21]. The activation of hedgehog signaling has also been associated with increased cell proliferation and tumor resistance in several solid tumors [22]. Here, we show that *KMT2A*-R and *MLLT10*-R were positively enriched in genes encoding members of the hedgehog signaling network (Fig. 2c), indicating that patients harboring these lesions might benefit from therapies with hedgehog inhibitors. While the mechanisms mediating epithelial to mesenchymal transition (EMT) have been widely studied in solid tumors, several lines of evidence indicate a critical role of EMT modulators in promoting leukemia cell motility and migration [23]. Our data demonstrate positive enrichment in genes associated with EMT in *KMT2A*-R, suggesting the need to further investigate the roles of EMT in T-ALL progression and resistance (Fig. 2c).

While our findings demonstrated that *KMT2A*-R share common biological networks with *MLLT10*-R [4], *KMT2A*-R also shared similar gene expression signatures with *KMT2A*-R in AML or BCP-ALL [5, 11, 12]. Our GSEA results demonstrate a strong enrichment for published data sets of genes differentially expressed in *KMT2A*-R leukemias (Additional file 8: Figure S3). We also identified signaling networks that could discriminate between *KMT2A*-R and *MLLT10*-R T-ALL. Compared to *KMT2A*-R, *MLLT10*-R showed upregulation in multiple receptor and/or tyrosine kinase-mediated pathways (e.g. *Met, TGFB, PYK2, ERBB, PDGFR*), for which FDA-approved inhibitors are available (Fig. 2d, Additional file 6: Table S5). These findings are of great interest considering that *MLLT10*-R were associated with T-ALL relapse [10]. In summary, this study reports findings for 12 T-ALL cases with *KMT2A*-R, which extends upon the observations by others [2, 5]. Overall, we have identified an extended repertoire of aberrant gene expression profiles in *KMT2A*-R and *MLLT10*-R T-ALL. These findings provide a mechanistic basis for additional pre-clinical testing in classes of therapeutic agents that may hold promise for high-risk T-ALL.

## Additional files

Additional file 1: **Figure S1A.** KMT2A-MLLT4 and KMT2A-MLLT1. (PDF 169 kb)

Additional file 2: **Table S1.** Probe sets for KMT2A-R and MLLT10R. (PDF 372 kb)

Additional file 3: **Table S2.** Validation of gene expression for KMT2A-R. (PDF 446 kb)

Additional file 4: **Table S3.** Validation of gene expression for MLLT10-R. (PDF 243 kb)

Additional file 5: **Table S4.** GSEA for gene ontology. (PDF 265 kb)

Additional file 6: **Table S5.** GSEA for canonical pathways. (PDF 276 kb)

Additional file 7: **Figure S2.** Genes positively enriched in MLLT10. (PDF 107 kb)

Additional file 8: **Figure S3.** KMT2A-R in B-ALL and AML. (PDF 231 kb)
