# Supplementary material for: Dysregulated transcriptional networks in KMT2A- and MLLT10-rearranged T-ALL
Source: Biomark Res. 2018 Aug 23;6:27. doi: 10.1186/s40364-018-0141-z (PMC6107954; doi:10.1186/s40364-018-0141-z)

Supplementary Figure S1. (A) Venn diagram for 330 probe sets related to commonly and exclusively differentially expressed genes in 3 T-ALL groups: *KMT2A*-R, *MLLT10*-R and Others (FDR ≤ 0.05). **(B)** Genes differentially expressed in T-ALL harboring *KMT2A-MLLT4* (n = 5) and *KMT2A-MLLT1* (n = 5) fusion transcripts.

A.

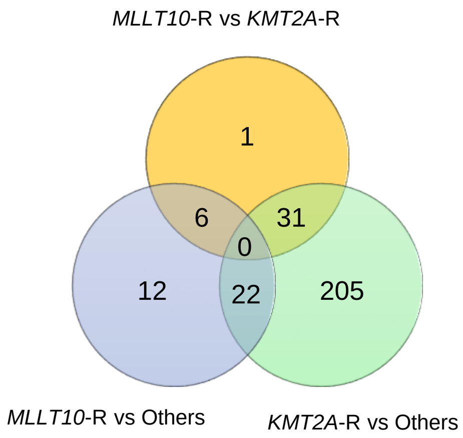

B.

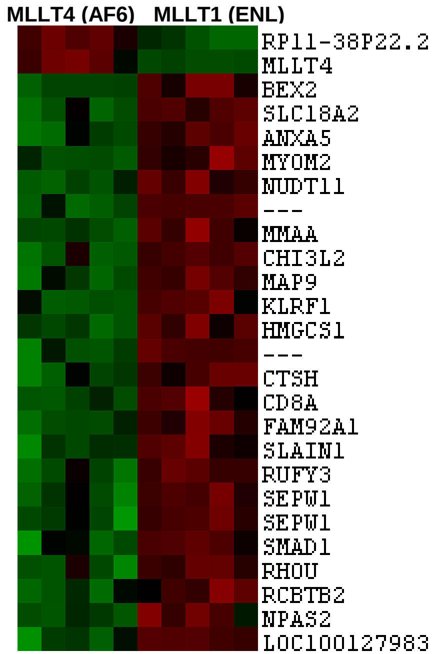

Supplement: Supplementary file 1 — Figure S1A. KMT2A-MLLT4 and KMT2A-MLLT1. (PDF 169 kb) [file 40364_2018_141_MOESM1_ESM.pdf]
