# Supplementary material for: Dysregulated transcriptional networks in KMT2A- and MLLT10-rearranged T-ALL
Source: Biomark Res. 2018 Aug 23;6:27. doi: 10.1186/s40364-018-0141-z (PMC6107954; doi:10.1186/s40364-018-0141-z)
Supplement: Supplementary file 2 — Table S1. Probe sets for KMT2A-R and MLLT10R. (PDF 372 kb) [file 40364_2018_141_MOESM2_ESM.pdf]

Supplementary Table S1. List of 330 probe sets differentially expressed in KMT2A-R and MLLT10-R T-ALL.

MLLT10-R vs KMT2A-R					
**Probe Set ID**	**Gene Symbol**	**Fold Chage**	**t**	**p-value**	**FDR**
203216_s_at	MYO6	-11.70	-8.01	2.21E-12	1.20E-07
236859_at	RUNX2	-4.85	-7.72	9.21E-12	2.51E-07
236858_s_at	RUNX2	-3.42	-7.59	1.73E-11	3.15E-07
203215_s_at	MYO6	-4.93	-7.14	1.52E-10	2.08E-06
226939_at	CPEB2	-10.63	-5.97	3.64E-08	0.000397252
240927_at	---	-3.24	-5.92	4.67E-08	0.000424446
240468_at	---	-4.26	-5.80	7.89E-08	0.000614591
210480_s_at	MYO6	-1.90	-5.31	6.74E-07	0.004405684
213908_at	WHAMMP2 /// WHAMMP3	-4.69	-5.29	7.27E-07	0.004405684
209763_at	CHRDL1	4.00	5.26	8.22E-07	0.00448206
228837_at	TCF4	-1.59	-5.17	1.23E-06	0.006088857
1557261_at	WHAMMP2 /// WHAMMP3	-2.41	-5.12	1.50E-06	0.00683231
244105_at	WHAMMP2 /// WHAMMP3	-1.50	-5.00	2.45E-06	0.010287413
214705_at	INADL	-2.52	-4.88	4.13E-06	0.015277057
1553145_at	TAPT1-AS1	-2.10	-4.87	4.20E-06	0.015277057
243617_at	ZNF827	-3.31	-4.83	4.99E-06	0.016887005
212382_at	TCF4	-5.11	-4.82	5.27E-06	0.016887005
232587_at	EML4	2.58	4.80	5.61E-06	0.016992083
226297_at	HIPK3	-3.13	-4.73	7.57E-06	0.02050316
213891_s_at	TCF4	-7.54	-4.72	7.77E-06	0.02050316
214772_at	KIAA1549L	-1.76	-4.71	7.90E-06	0.02050316
228116_at	---	4.08	4.69	8.68E-06	0.02150266
222146_s_at	TCF4	-4.44	-4.65	1.01E-05	0.023948246
212387_at	TCF4	-5.04	-4.64	1.06E-05	0.023959455
243618_s_at	ZNF827	-3.74	-4.61	1.21E-05	0.026329084
235479_at	CPEB2	-2.32	-4.59	1.29E-05	0.026975147
242881_x_at	LOC100506303 /// LOC100(	8.08	4.58	1.34E-05	0.027026521
206506_s_at	SUPT3H	-2.59	-4.55	1.53E-05	0.029778113
203753_at	TCF4	-5.44	-4.51	1.78E-05	0.033500853
212386_at	TCF4	-4.56	-4.50	1.87E-05	0.033982865
1553808_a_at	NKX2-3	1.79	4.47	2.09E-05	0.036713473
215163_at	---	-3.64	-4.44	2.30E-05	0.038003673
210993_s_at	SMAD1	-5.57	-4.44	2.30E-05	0.038003673
1556739_at	GOLGA8I	-1.66	-4.41	2.58E-05	0.041437598
209871_s_at	APBA2	-2.72	-4.40	2.74E-05	0.041558569
204304_s_at	PROM1	-20.73	-4.40	2.74E-05	0.041558569
231374_at	---	2.07	4.38	2.94E-05	0.04338079
202263_at	CYB5R1	-1.77	-4.36	3.17E-05	0.045502391
**KMT2A-R vs Others**					
203216_s_at	MYO6	12.28	12.05	3.86E-21	1.47E-16
236859_at	RUNX2	5.27	11.98	5.40E-21	1.47E-16
236858_s_at	RUNX2	3.54	11.49	6.12E-20	1.11E-15

203215_s_at	MYO6	4.40	9.78	3.16E-16	3.94E-12
1557261_at	WHAMMP2 /// WHAMMP3	3.12	9.75	3.62E-16	3.94E-12
235753_at	HOXA7	3.37	9.61	7.53E-16	6.84E-12
226939_at	CPEB2	12.25	9.33	3.03E-15	2.36E-11
235479_at	CPEB2	3.06	9.00	1.57E-14	1.07E-10
235521_at	HOXA3	6.13	8.81	4.11E-14	2.49E-10
213908_at	WHAMMP2 /// WHAMMP3	5.71	8.79	4.58E-14	2.49E-10
213844_at	HOXA5	11.64	8.73	6.09E-14	3.02E-10
1559266_s_at	SKIDA1	3.65	8.62	1.06E-13	4.83E-10
240927_at	---	3.19	8.59	1.23E-13	5.15E-10
240468_at	---	4.25	8.53	1.66E-13	6.44E-10
206847_s_at	HOXA7	3.40	8.41	3.05E-13	1.11E-09
209905_at	HOXA10-HOXA9 /// HOXA9	33.09	8.36	4.04E-13	1.38E-09
214651_s_at	HOXA10-HOXA9 /// HOXA9	37.44	7.97	2.70E-12	8.65E-09
210480_s_at	MYO6	1.92	7.94	3.22E-12	9.54E-09
228837_at	TCF4	1.63	7.93	3.32E-12	9.54E-09
206289_at	HOXA4	1.91	7.90	3.91E-12	1.07E-08
206506_s_at	SUPT3H	3.06	7.88	4.27E-12	1.11E-08
225627_s_at	CACHD1	2.27	7.38	4.80E-11	1.19E-07
227195_at	ZNF503	5.78	7.34	6.07E-11	1.44E-07
215204_at	---	3.35	7.26	8.68E-11	1.97E-07
229290_at	DAPL1	2.82	7.25	9.40E-11	2.05E-07
244105_at	WHAMMP2 /// WHAMMP3	1.48	7.07	2.23E-10	4.67E-07
203961_at	NEBL	2.38	6.74	1.04E-09	2.09E-06
214772_at	KIAA1549L	1.73	6.74	1.07E-09	2.09E-06
203733_at	DEXI	2.17	6.69	1.35E-09	2.54E-06
243605_at	---	2.94	6.65	1.62E-09	2.94E-06
208557_at	HOXA6	2.01	6.60	2.00E-09	3.51E-06
233792_at	---	3.40	6.52	2.91E-09	4.96E-06
1560999_a_at	---	2.92	6.51	3.04E-09	5.01E-06
236442_at	DPF3	2.98	6.49	3.33E-09	5.33E-06
222146_s_at	TCF4	4.07	6.46	3.97E-09	6.18E-06
213147_at	HOXA10	2.72	6.42	4.68E-09	7.08E-06
235486_at	KIAA1549L	2.19	6.40	5.17E-09	7.61E-06
1559265_at	SKIDA1	1.48	6.37	5.89E-09	8.45E-06
213150_at	HOXA10	7.65	6.36	6.19E-09	8.66E-06
214705_at	INADL	2.26	6.35	6.57E-09	8.95E-06
221458_at	HTR1F	1.99	6.33	7.14E-09	9.50E-06
239503_at	C10orf114	1.62	6.31	7.76E-09	9.87E-06
229823_at	RIMS2	3.33	6.31	7.79E-09	9.87E-06
1555923_a_at	C10orf114	1.67	6.30	8.10E-09	1.00E-05
212382_at	TCF4	4.24	6.29	8.52E-09	1.03E-05
236565_s_at	LARP6	1.78	6.26	9.97E-09	1.18E-05
238532_at	DPF3	3.70	6.23	1.13E-08	1.31E-05
221631_at	CACNA1I	1.93	6.19	1.33E-08	1.51E-05
204304_s_at	PROM1	17.98	6.18	1.42E-08	1.57E-05
208015_at	SMAD1	2.20	6.07	2.30E-08	2.51E-05

212387_at	TCF4	4.15	6.01	3.01E-08	3.22E-05
202289_s_at	TACC2	1.89	5.98	3.60E-08	3.77E-05
220588_at	BCAS4	1.66	5.95	3.94E-08	4.05E-05
1553145_at	TAPT1-AS1	1.85	5.95	4.04E-08	4.07E-05
219988_s_at	RNF220	1.83	5.93	4.45E-08	4.41E-05
203753_at	TCF4	4.48	5.89	5.21E-08	5.08E-05
212386_at	TCF4	3.85	5.88	5.41E-08	5.17E-05
206137_at	RIMS2	1.93	5.88	5.61E-08	5.27E-05
243768_at	---	2.47	5.78	8.65E-08	7.99E-05
223681_s_at	INADL	2.03	5.73	1.08E-07	9.81E-05
213891_s_at	TCF4	5.26	5.72	1.15E-07	0.000102536
1553118_at	THEM4	1.77	5.67	1.39E-07	0.000122302
208343_s_at	NR5A2	1.91	5.65	1.56E-07	0.000133052
211106_at	SUPT3H	2.62	5.65	1.56E-07	0.000133052
226297_at	HIPK3	2.51	5.63	1.71E-07	0.000143347
205889_s_at	JAKMIP2	1.51	5.59	2.04E-07	0.000167512
1556739_at	GOLGA8I	1.54	5.58	2.06E-07	0.000167512
215163_at	---	3.00	5.57	2.17E-07	0.00017361
212385_at	TCF4	2.77	5.54	2.50E-07	0.000197795
217585_at	NEBL	1.76	5.53	2.59E-07	0.000201603
210993_s_at	SMAD1	4.20	5.48	3.26E-07	0.000250249
232645_at	LOC153684	1.61	5.44	3.84E-07	0.000291054
209871_s_at	APBA2	2.29	5.36	5.35E-07	0.000394572
217520_x_at	LOC283683 /// LOC646278	4.10	5.36	5.37E-07	0.000394572
238798_at	TAPT1	1.88	5.36	5.43E-07	0.000394572
206999_at	IL12RB2	2.18	5.34	5.79E-07	0.000415504
209199_s_at	MEF2C	4.74	5.34	5.91E-07	0.000418146
1559315_s_at	SOCS2-AS1	2.60	5.31	6.79E-07	0.000474142
204647_at	HOMER3	1.66	5.30	6.93E-07	0.000477973
1555270_a_at	WFS1	1.37	5.30	7.02E-07	0.000478607
221349_at	VPREB1	4.83	5.29	7.19E-07	0.000484097
1559477_s_at	MEIS1	3.58	5.29	7.38E-07	0.000490668
232231_at	RUNX2	11.93	5.28	7.47E-07	0.000490668
204069_at	MEIS1	4.11	5.27	8.08E-07	0.000524145
37802_r_at	FAM63B	1.41	5.25	8.59E-07	0.000551113
221654_s_at	USP3	-2.98	-5.24	8.94E-07	0.000566537
214790_at	SENP6	4.49	5.18	1.15E-06	0.000719977
205327_s_at	ACVR2A	1.44	5.17	1.22E-06	0.000754857
204361_s_at	SKAP2	3.07	5.16	1.24E-06	0.000759634
218597_s_at	CISD1	-2.43	-5.13	1.42E-06	0.000858703
239963_at	---	2.87	5.11	1.56E-06	0.000935743
228416_at	ACVR2A	1.58	5.11	1.59E-06	0.000941977
203962_s_at	NEBL	1.82	5.10	1.62E-06	0.000941977
227877_at	ANXA2R	2.43	5.10	1.62E-06	0.000941977
202967_at	GSTA4	1.98	5.10	1.65E-06	0.000946076
237680_at	---	1.68	5.09	1.70E-06	0.000963206
227592_at	ALDH16A1	1.41	5.09	1.72E-06	0.000968498

203349_s_at	ETV5	3.59	5.07	1.84E-06	0.001025036
205448_s_at	MAP3K12	1.33	5.06	1.92E-06	0.001059239
242598_at	---	3.04	5.02	2.29E-06	0.00124876
225752_at	NIPA1	2.68	5.01	2.34E-06	0.001262535
210174_at	NR5A2	1.95	5.00	2.45E-06	0.001308159
228855_at	NUDT7	1.85	4.98	2.64E-06	0.001398857
234994_at	TMEM200A	2.15	4.98	2.70E-06	0.001415254
212265_at	QKI	-2.92	-4.97	2.79E-06	0.001447217
235291_s_at	FLJ32255	2.10	4.95	3.03E-06	0.001555899
206335_at	GALNS	1.51	4.91	3.65E-06	0.001857477
211685_s_at	NCALD	1.48	4.90	3.69E-06	0.001862624
242414_at	QPRT	1.79	4.89	3.94E-06	0.001953288
209870_s_at	APBA2	1.65	4.89	3.94E-06	0.001953288
226140_s_at	OTUD1	3.05	4.87	4.15E-06	0.002039773
205402_x_at	PRSS2	3.68	4.86	4.39E-06	0.002138117
203836_s_at	MAP3K5	2.05	4.85	4.63E-06	0.002231891
205349_at	GNA15	-2.90	-4.82	5.19E-06	0.002482394
242461_at	---	1.37	4.81	5.46E-06	0.002587769
235173_at	MBNL1-AS1	1.34	4.79	5.81E-06	0.002718792
46665_at	SEMA4C	1.66	4.79	5.84E-06	0.002718792
221696_s_at	STYK1	1.26	4.78	6.08E-06	0.002807472
218409_s_at	DNAJC1	2.23	4.77	6.29E-06	0.002880685
216048_s_at	RHOBTB3	1.69	4.74	7.11E-06	0.003229394
214691_x_at	FAM63B	1.60	4.74	7.22E-06	0.003252724
215164_at	---	1.91	4.73	7.42E-06	0.003316773
205518_s_at	CMAHP	-4.24	-4.72	7.71E-06	0.003415655
240570_at	INADL	1.33	4.70	8.33E-06	0.003661292
210033_s_at	SPAG6	2.50	4.69	8.74E-06	0.003794438
213428_s_at	COL6A1	2.03	4.69	8.77E-06	0.003794438
200984_s_at	CD59	2.24	4.67	9.39E-06	0.004002591
236395_at	---	4.44	4.67	9.40E-06	0.004002591
244230_at	---	1.35	4.67	9.57E-06	0.004045434
239578_at	---	1.57	4.66	9.75E-06	0.00408802
212263_at	QKI	-4.40	-4.66	1.00E-05	0.004164931
225710_at	GNB4	-3.97	-4.62	1.17E-05	0.004816511
204981_at	SLC22A18	1.40	4.60	1.25E-05	0.005120026
229393_at	L3MBTL3	2.62	4.59	1.32E-05	0.005376412
226764_at	ZNF827	2.53	4.56	1.46E-05	0.00588912
206746_at	BFSP1	1.17	4.56	1.48E-05	0.005935402
228221_at	SLC44A3	1.82	4.55	1.51E-05	0.005992953
209647_s_at	SOCS5	-1.67	-4.53	1.66E-05	0.006560549
225639_at	SKAP2	4.31	4.51	1.76E-05	0.00683016
233280_at	---	1.53	4.51	1.77E-05	0.00683016
243618_s_at	ZNF827	2.40	4.51	1.78E-05	0.00683016
234107_s_at	DTD1	2.44	4.51	1.78E-05	0.00683016
213169_at	SEMA5A	1.44	4.49	1.91E-05	0.007238218
223836_at	FGFBP2	2.47	4.49	1.91E-05	0.007238218

213693_s_at	MUC1	1.37	4.48	1.98E-05	0.007457255
204675_at	SRD5A1	2.02	4.46	2.19E-05	0.008126061
210032_s_at	SPAG6	1.61	4.46	2.19E-05	0.008126061
240180_at	---	1.81	4.45	2.24E-05	0.008245656
242172_at	---	2.53	4.44	2.29E-05	0.008377552
209200_at	MEF2C	2.71	4.43	2.39E-05	0.008674413
218380_at	LOC728392	2.38	4.43	2.41E-05	0.008679631
243617_at	ZNF827	2.11	4.43	2.42E-05	0.008679631
210542_s_at	SLCO3A1	1.39	4.43	2.47E-05	0.008766313
229253_at	THEM4	1.91	4.42	2.48E-05	0.008766313
243492_at	THEM4	1.39	4.41	2.62E-05	0.009209529
232629_at	PROK2	2.36	4.41	2.64E-05	0.009210533
213644_at	CEP112	1.40	4.39	2.86E-05	0.009849459
222111_at	FAM63B	2.63	4.39	2.87E-05	0.009849459
214752_x_at	FLNA	1.59	4.39	2.88E-05	0.009849459
1560018_at	ARPP21	1.77	4.38	2.89E-05	0.009849459
215307_at	ZNF529	-2.52	-4.38	2.96E-05	0.010019553
219225_at	PGBD5	1.46	4.37	3.01E-05	0.010127668
211056_s_at	SRD5A1	1.42	4.37	3.04E-05	0.01016145
206361_at	PTGDR2	1.39	4.35	3.28E-05	0.010891796
241936_x_at	---	1.44	4.35	3.36E-05	0.011097313
235957_at	GRIP1	1.74	4.32	3.76E-05	0.012276055
230102_at	ETV5	2.00	4.31	3.78E-05	0.012276055
200983_x_at	CD59	2.00	4.31	3.78E-05	0.012276055
211555_s_at	GUCY1B3	1.60	4.31	3.85E-05	0.012423472
1556842_at	LOC286087	2.12	4.30	4.04E-05	0.012898335
229636_at	---	1.26	4.30	4.05E-05	0.012898335
205578_at	ROR2	1.25	4.29	4.12E-05	0.013063086
227399_at	VGLL3	1.92	4.28	4.30E-05	0.013556911
207463_x_at	PRSS2 /// PRSS3	2.08	4.26	4.64E-05	0.014548924
219746_at	DPF3	1.54	4.26	4.68E-05	0.014582277
202970_at	DYRK2	1.64	4.26	4.74E-05	0.014667249
202255_s_at	SIPA1L1	1.46	4.25	4.81E-05	0.014807474
204362_at	SKAP2	3.26	4.24	5.00E-05	0.015301537
216470_x_at	PRSS2	2.34	4.22	5.42E-05	0.0165062
241782_at	NEBL	1.30	4.21	5.55E-05	0.016769611
220102_at	FOXL2	1.84	4.21	5.57E-05	0.016769611
227798_at	SMAD1	3.92	4.21	5.61E-05	0.016804297
236901_at	---	1.28	4.19	5.98E-05	0.01780632
1560662_s_at	WHAMMP2 /// WHAMMP3	2.10	4.19	6.11E-05	0.018090705
232051_at	CCDC102A	1.27	4.19	6.14E-05	0.018090705
209581_at	PLA2G16	1.52	4.18	6.17E-05	0.018090705
203348_s_at	ETV5	1.75	4.18	6.31E-05	0.018404358
225280_x_at	ARSD	1.35	4.17	6.50E-05	0.018831384
204044_at	QPRT	1.67	4.16	6.66E-05	0.019137914
210959_s_at	SRD5A1	1.41	4.16	6.70E-05	0.019137914
211382_s_at	TACC2	1.44	4.16	6.71E-05	0.019137914

226981_at	KMT2A	-1.97	-4.16	6.82E-05	0.019367905
213737_x_at	GOLGA8I /// GOLGA8O	3.26	4.15	6.93E-05	0.019569442
228748_at	CD59	1.39	4.15	7.04E-05	0.019770606
208127_s_at	SOCS5	-2.33	-4.14	7.37E-05	0.020601592
232597_x_at	SCAF11	1.56	4.13	7.54E-05	0.02097775
234423_x_at	LOC100996255	1.50	4.12	7.79E-05	0.021540416
1560758_at	---	1.31	4.12	7.97E-05	0.021947588
238498_at	OTTHUMG00000175943 //	1.57	4.11	8.12E-05	0.022244763
209648_x_at	SOCS5	-2.49	-4.11	8.19E-05	0.022313535
239296_at	---	1.60	4.11	8.30E-05	0.022497752
231902_at	ZNF827	1.57	4.10	8.41E-05	0.022698506
205229_s_at	COCH	1.74	4.10	8.51E-05	0.022852112
201997_s_at	SPEN	-1.95	-4.09	8.66E-05	0.023132725
200985_s_at	CD59	2.88	4.08	8.95E-05	0.023788199
1560512_at	---	1.45	4.08	9.08E-05	0.024026168
226971_at	CCDC136	1.35	4.07	9.47E-05	0.024927287
220488_s_at	BCAS3	1.37	4.07	9.58E-05	0.025092438
232298_at	MBNL1-AS1	1.17	4.04	0.000103619	0.027022287
226873_at	FAM63B	2.88	4.04	0.00010555	0.02739474
227769_at	GPR27	2.29	4.03	0.000108062	0.027913717
216373_at	TAPT1	2.65	4.03	0.00011083	0.028482951
235758_at	PNMA6A /// PNMA6B /// P	1.47	4.03	0.000111311	0.028482951
202975_s_at	RHOBTB3	2.24	4.02	0.000112057	0.028539993
1568589_at	---	1.51	4.02	0.000114859	0.029117484
1555963_x_at	B3GNT7	1.31	4.01	0.000117638	0.02968411
205572_at	ANGPT2	1.55	4.00	0.000121949	0.030630088
229461_x_at	NEGR1	3.32	3.99	0.000124223	0.031058046
236008_at	LOC100128909	1.35	3.98	0.000132662	0.033016458
239842_x_at	---	1.36	3.97	0.000135457	0.033558823
205471_s_at	DACH1	2.08	3.97	0.000136785	0.033734564
202908_at	WFS1	1.38	3.97	0.000138374	0.033917778
1554703_at	ARHGEF10	1.56	3.96	0.000138773	0.033917778
242055_at	PSMG4	1.93	3.96	0.000140209	0.033923779
214930_at	SLITRK5	1.50	3.96	0.000140277	0.033923779
238850_at	LINC00461 /// MIR9-2	2.29	3.96	0.00014098	0.033923779
215802_at	---	1.58	3.96	0.000141287	0.033923779
243049_at	---	1.45	3.95	0.00014366	0.034324108
202254_at	SIPA1L1	1.73	3.95	0.000144214	0.034324108
214535_s_at	ADAMTS2	1.26	3.92	0.000160557	0.037956425
214639_s_at	HOXA1	1.35	3.92	0.000161187	0.037956425
228412_at	LOC643072	1.33	3.92	0.000161936	0.037956425
209572_s_at	EED	-2.02	-3.92	0.000162261	0.037956425
240321_at	---	1.52	3.92	0.000164647	0.03830306
218831_s_at	FCGRT	1.55	3.92	0.000165148	0.03830306
203870_at	USP46	1.70	3.91	0.00016625	0.038395182
210304_at	PDE6B	1.29	3.90	0.000173966	0.040007719
221610_s_at	STAP2	-1.28	-3.89	0.000179237	0.041046875

213421_x_at	PRSS2 /// PRSS3	2.00	3.89	0.000181869	0.041442685
219860_at	LY6G5C	1.25	3.89	0.000182487	0.041442685
225949_at	NRBP2	1.75	3.88	0.000186442	0.042165293
215197_at	---	1.31	3.88	0.000187591	0.042249943
237058_x_at	SLC6A13	1.93	3.88	0.000189751	0.042560388
225286_at	ARSD	1.39	3.88	0.000190771	0.042613782
244480_at	---	1.33	3.87	0.000193841	0.043122886
226415_at	VAT1L	1.47	3.87	0.000196142	0.043457388
220030_at	STYK1	1.28	3.86	0.000199783	0.044084929
226311_at	ADAMTS2	1.38	3.86	0.000200996	0.044173828
231035_s_at	OTUD1	1.54	3.86	0.000204599	0.044784983
1555842_at	CYTH2	1.53	3.85	0.000211807	0.046084642
236632_at	HHIP-AS1	2.20	3.84	0.000213761	0.046084642
211707_s_at	IQCB1	-1.55	-3.84	0.000213834	0.046084642
204671_s_at	ANKRD6	1.61	3.84	0.000213919	0.046084642
1562194_at	---	1.81	3.84	0.000215744	0.046294852
1566903_at	---	1.31	3.84	0.000216882	0.04635669
205488_at	GZMA	3.78	3.84	0.000220249	0.046892362
205826_at	MYOM2	2.84	3.82	0.000229768	0.04872876
218376_s_at	MICAL1	2.13	3.82	0.000236327	0.049925552
	**MLLT10-R vs Others**				
213844_at	HOXA5	25.20	9.58	8.52E-16	4.64E-11
209763_at	CHRDL1	7.06	9.13	8.54E-15	2.33E-10
235521_at	HOXA3	8.55	8.71	6.86E-14	1.25E-09
1559477_s_at	MEIS1	8.04	7.22	1.08E-10	1.22E-06
235753_at	HOXA7	2.98	7.21	1.12E-10	1.22E-06
214639_s_at	HOXA1	1.93	7.15	1.47E-10	1.33E-06
204069_at	MEIS1	9.85	7.11	1.79E-10	1.39E-06
206289_at	HOXA4	1.97	6.90	5.01E-10	3.41E-06
1559266_s_at	SKIDA1	3.38	6.78	8.71E-10	5.27E-06
209905_at	HOXA10-HOXA9 /// HOXA9	25.73	6.47	3.65E-09	1.99E-05
214651_s_at	HOXA10-HOXA9 /// HOXA9	33.07	6.43	4.53E-09	2.25E-05
242172_at	---	4.93	6.38	5.78E-09	2.62E-05
206847_s_at	HOXA7	2.93	6.16	1.54E-08	6.45E-05
243003_at	---	3.53	6.04	2.63E-08	0.000102291
210033_s_at	SPAG6	4.04	5.97	3.67E-08	0.000131302
227949_at	PHACTR3	2.39	5.96	3.92E-08	0.000131302
205600_x_at	HOXB5	1.46	5.95	4.10E-08	0.000131302
210032_s_at	SPAG6	2.13	5.89	5.35E-08	0.000161996
228116_at	---	4.12	5.81	7.40E-08	0.000212321
1553808_a_at	NKX2-3	1.81	5.63	1.70E-07	0.000462977
242881_x_at	LOC100506303 /// LOC100(	7.94	5.59	1.98E-07	0.000513061
237600_at	---	2.84	5.56	2.29E-07	0.000566645
242216_at	---	7.79	5.39	4.84E-07	0.001146214
235149_at	PGM2L1	1.48	5.31	6.78E-07	0.001539902
232587_at	EML4	2.31	5.21	1.02E-06	0.002213083

227195_at	ZNF503	4.35	5.13	1.44E-06	0.003021053
231374_at	---	1.99	5.10	1.66E-06	0.00335235
202265_at	BMI1 /// COMMD3-BMI1	4.14	4.89	3.95E-06	0.007510162
208557_at	HOXA6	1.86	4.88	4.00E-06	0.007510162
213150_at	HOXA10	6.47	4.87	4.17E-06	0.007582198
1555923_a_at	C10orf114	1.58	4.73	7.35E-06	0.012914977
205408_at	MLLT10	1.85	4.71	8.18E-06	0.013927419
219988_s_at	RNF220	1.77	4.66	1.00E-05	0.016557975
229667_s_at	HOXB8	1.51	4.54	1.61E-05	0.025784551
1559265_at	SKIDA1	1.40	4.52	1.70E-05	0.026507439
225992_at	MLLT10	2.55	4.48	1.96E-05	0.029702992
213147_at	HOXA10	2.31	4.47	2.04E-05	0.030068449
228708_at	RAB27B	4.21	4.45	2.24E-05	0.032191537
1569448_at	PGM2L1	2.00	4.36	3.22E-05	0.045032336
212265_at	QKI	-3.07	-4.34	3.41E-05	0.046453564
