# Supplementary material for: Dysregulated transcriptional networks in KMT2A- and MLLT10-rearranged T-ALL
Source: Biomark Res. 2018 Aug 23;6:27. doi: 10.1186/s40364-018-0141-z (PMC6107954; doi:10.1186/s40364-018-0141-z)
Supplement: Supplementary file 3 — Table S2. Validation of gene expression for KMT2A-R. (PDF 446 kb) [file 40364_2018_141_MOESM3_ESM.pdf]

Supplementary Table S2. Gene expression profiling for KMT2A-R, MLLT10-R in a molecular interrogation cohort of 100 T-ALL drawn from COG AALL0434 study and the validation cohort of Soulier et al. (2005). Differentially expressed genes in MLL-R T-ALL vs Others ("Others" reflecting cases that do not have either KMT2A-R or MLLT10-R.), MLLT10-R vs Others, MLLT10-R10 vs. KMT2A-R.

KMT2A-R vs Others

Probe Set ID	Gene Symbol	COG Data				Soulier's Data				Validated
		Fold Chage	t	p-value	FDR	Fold Chage	t	p-value	FDR	
203216_s_at	MYO6	12.28	12.05	3.86E-21	1.47E-16	19.39	76.92	4.32E-81	5.96E-79	Yes
236859_at	RUNX2	5.27	11.98	5.40E-21	1.47E-16					
236858_s_at	RUNX2	3.54	11.49	6.12E-20	1.11E-15					
203215_s_at	MYO6	4.40	9.78	3.16E-16	3.94E-12	1.91	11.43	6.06E-19	1.04E-17	Yes
1557261_at	WHAMMP2 /// WHAMMP3	3.12	9.75	3.62E-16	3.94E-12					
235753_at	HOXA7	3.37	9.61	7.53E-16	6.84E-12					
226939_at	CPEB2	12.25	9.33	3.03E-15	2.36E-11					
235479_at	CPEB2	3.06	9.00	1.57E-14	1.07E-10					
235521_at	HOXA3	6.13	8.81	4.11E-14	2.49E-10					
213908_at	WHAMMP2 /// WHAMMP3	5.71	8.79	4.58E-14	2.49E-10	3.51	13.30	1.38E-22	3.82E-21	Yes
213844_at	HOXA5	11.64	8.73	6.09E-14	3.02E-10	6.24	4.34	3.84E-05	0.0001203	Yes
1559266_s_at	SKIDA1	3.65	8.62	1.06E-13	4.83E-10					
240927_at	---	3.19	8.59	1.23E-13	5.15E-10					
240468_at	---	4.25	8.53	1.66E-13	6.44E-10					
206847_s_at	HOXA7	3.40	8.41	3.05E-13	1.11E-09	1.36	3.33	0.0012926	0.00302336	Yes
209905_at	HOXA10-HOXA9 /// HOXA9 /// MIR196B	33.09	8.36	4.04E-13	1.38E-09	9.30	5.00	3.00E-06	1.22E-05	Yes
214651_s_at	HOXA10-HOXA9 /// HOXA9 /// MIR196B	37.44	7.97	2.70E-12	8.65E-09	23.06	4.89	4.68E-06	1.85E-05	Yes
210480_s_at	MYO6	1.92	7.94	3.22E-12	9.54E-09	3.96	16.86	5.26E-29	2.42E-27	Yes
228837_at	TCF4	1.63	7.93	3.32E-12	9.54E-09					
206289_at	HOXA4	1.91	7.90	3.91E-12	1.07E-08	1.44	3.74	0.00033139	0.0008469	Yes
206506_s_at	SUPT3H	3.06	7.88	4.27E-12	1.11E-08	4.44	7.35	1.08E-10	6.77E-10	Yes
225627_s_at	CACHD1	2.27	7.38	4.80E-11	1.19E-07					
227195_at	ZNF503	5.78	7.34	6.07E-11	1.44E-07					
215204_at	---	3.35	7.26	8.68E-11	1.97E-07	3.78	7.36	1.03E-10	6.74E-10	Yes
229290_at	DAPL1	2.82	7.25	9.40E-11	2.05E-07					
244105_at	WHAMMP2 /// WHAMMP3	1.48	7.07	2.23E-10	4.67E-07					
203961_at	NEBL	2.38	6.74	1.04E-09	2.09E-06	6.66	10.07	3.27E-16	4.51E-15	Yes
214772_at	KIAA1549L	1.73	6.74	1.07E-09	2.09E-06	1.91	11.53	3.92E-19	7.73E-18	Yes
203733_at	DEXI	2.17	6.69	1.35E-09	2.54E-06	1.34	2.17	0.03256224	0.05479987	No
243605_at	---	2.94	6.65	1.62E-09	2.94E-06					
208557_at	HOXA6	2.01	6.60	2.00E-09	3.51E-06	1.33	2.94	0.00422875	0.00845751	Yes
233792_at	---	3.40	6.52	2.91E-09	4.96E-06					
1560999_a_at	---	2.92	6.51	3.04E-09	5.01E-06					
236442_at	DPF3	2.98	6.49	3.33E-09	5.33E-06					
222146_s_at	TCF4	4.07	6.46	3.97E-09	6.18E-06	9.53	9.48	5.17E-15	5.49E-14	Yes
213147_at	HOXA10	2.72	6.42	4.68E-09	7.08E-06	2.18	2.32	0.02268423	0.03962562	Yes
235486_at	KIAA1549L	2.19	6.40	5.17E-09	7.61E-06					
1559265_at	SKIDA1	1.48	6.37	5.89E-09	8.45E-06					
213150_at	HOXA10	7.65	6.36	6.19E-09	8.66E-06	5.07	3.51	0.00071312	0.00169672	Yes
214705_at	INADL	2.26	6.35	6.57E-09	8.95E-06	1.06	1.15	0.25444382	0.31351114	No

221458_at	HTR1F	1.99	6.33	7.14E-09	9.50E-06	2.43	9.63	2.55E-15	2.93E-14	Yes
239503_at	C10orf114	1.62	6.31	7.76E-09	9.87E-06					
229823_at	RIMS2	3.33	6.31	7.79E-09	9.87E-06					
1555923_a_at	C10orf114	1.67	6.30	8.10E-09	1.00E-05					
212382_at	TCF4	4.24	6.29	8.52E-09	1.03E-05	7.10	13.67	2.76E-23	9.52E-22	Yes
236565_s_at	LARP6	1.78	6.26	9.97E-09	1.18E-05					
238532_at	DPF3	3.70	6.23	1.13E-08	1.31E-05					
221631_at	CACNA1I	1.93	6.19	1.33E-08	1.51E-05	1.68	4.39	3.22E-05	0.00010578	Yes
204304_s_at	PROM1	17.98	6.18	1.42E-08	1.57E-05	82.89	7.29	1.42E-10	8.15E-10	Yes
208015_at	SMAD1	2.20	6.07	2.30E-08	2.51E-05	2.21	5.44	4.91E-07	2.12E-06	Yes
212387_at	TCF4	4.15	6.01	3.01E-08	3.22E-05	10.52	8.69	2.07E-13	2.04E-12	Yes
202289_s_at	TACC2	1.89	5.98	3.60E-08	3.77E-05	1.14	1.78	0.07797512	0.11326912	No
220588_at	BCAS4	1.66	5.95	3.94E-08	4.05E-05	1.22	1.88	0.06369916	0.09554875	No
1553145_at	TAPT1-AS1	1.85	5.95	4.04E-08	4.07E-05					
219988_s_at	RNF220	1.83	5.93	4.45E-08	4.41E-05	2.15	7.97	6.04E-12	4.63E-11	Yes
203753_at	TCF4	4.48	5.89	5.21E-08	5.08E-05	5.33	8.46	6.09E-13	5.61E-12	Yes
212386_at	TCF4	3.85	5.88	5.41E-08	5.17E-05	19.30	8.09	3.42E-12	2.95E-11	Yes
206137_at	RIMS2	1.93	5.88	5.61E-08	5.27E-05	1.20	2.10	0.03855877	0.06334656	No
243768_at	---	2.47	5.78	8.65E-08	7.99E-05					
223681_s_at	INADL	2.03	5.73	1.08E-07	9.81E-05					
213891_s_at	TCF4	5.26	5.72	1.15E-07	0.000103	16.23	10.98	4.75E-18	7.29E-17	Yes
1553118_at	THEM4	1.77	5.67	1.39E-07	0.000122					
208343_s_at	NR5A2	1.91	5.65	1.56E-07	0.000133	-1.06	-0.34	0.73801617	0.77904883	No
211106_at	SUPT3H	2.62	5.65	1.56E-07	0.000133	2.54	4.83	5.95E-06	2.22E-05	Yes
226297_at	HIPK3	2.51	5.63	1.71E-07	0.000143					
205889_s_at	JAKMIP2	1.51	5.59	2.04E-07	0.000168	1.21	1.95	0.055038	0.08590809	No
1556739_at	GOLGA8I	1.54	5.58	2.06E-07	0.000168					
215163_at	---	3.00	5.57	2.17E-07	0.000174	1.26	2.46	0.01589357	0.02811938	Yes
212385_at	TCF4	2.77	5.54	2.50E-07	0.000198	4.16	12.97	5.99E-22	1.38E-20	Yes
217585_at	NEBL	1.76	5.53	2.59E-07	0.000202	1.77	8.04	4.43E-12	3.60E-11	Yes
210993_s_at	SMAD1	4.20	5.48	3.26E-07	0.00025	4.31	4.35	3.67E-05	0.00011788	Yes
232645_at	LOC153684	1.61	5.44	3.84E-07	0.000291					
209871_s_at	APBA2	2.29	5.36	5.35E-07	0.000395	1.15	0.75	0.4581292	0.51399861	No
217520_x_at	LOC283683 /// LOC646278	4.10	5.36	5.37E-07	0.000395	2.64	3.85	0.00023039	0.00061142	Yes
238798_at	TAPT1	1.88	5.36	5.43E-07	0.000395					
206999_at	IL12RB2	2.18	5.34	5.79E-07	0.000416	1.01	0.06	0.94897939	0.9608047	No
209199_s_at	MEF2C	4.74	5.34	5.91E-07	0.000418	4.66	3.13	0.00235248	0.00515304	Yes
1559315_s_at	SOCS2-AS1	2.60	5.31	6.79E-07	0.000474					
204647_at	HOMER3	1.66	5.30	6.93E-07	0.000478	1.54	3.57	0.00058163	0.0014333	Yes
1555270_a_at	WFS1	1.37	5.30	7.02E-07	0.000479					
221349_at	VPREB1	4.83	5.29	7.19E-07	0.000484	5.95	3.67	0.00041577	0.0010432	Yes
1559477_s_at	MEIS1	3.58	5.29	7.38E-07	0.000491					
232231_at	RUNX2	11.93	5.28	7.47E-07	0.000491					
204069_at	MEIS1	4.11	5.27	8.08E-07	0.000524	4.45	6.21	1.78E-08	8.18E-08	Yes
37802_r_at	FAM63B	1.41	5.25	8.59E-07	0.000551	1.39	6.45	6.18E-09	3.05E-08	Yes
221654_s_at	USP3	-2.98	-5.24	8.94E-07	0.000567	-1.65	-1.66	0.10092683	0.14212145	No
214790_at	SENP6	4.49	5.18	1.15E-06	0.00072	15.79	17.68	2.20E-30	1.52E-28	Yes
205327_s_at	ACVR2A	1.44	5.17	1.22E-06	0.000755	1.14	1.47	0.14397287	0.18743637	No
204361_s_at	SKAP2	3.07	5.16	1.24E-06	0.00076	2.25	3.98	0.00014335	0.00040552	Yes
218597_s_at	CISD1	-2.43	-5.13	1.42E-06	0.000859	-2.55	-3.26	0.00160989	0.00370275	Yes
239963_at	---	2.87	5.11	1.56E-06	0.000936					
228416_at	ACVR2A	1.58	5.11	1.59E-06	0.000942					
203962_s_at	NEBL	1.82	5.10	1.62E-06	0.000942	6.29	10.02	4.19E-16	5.26E-15	Yes
227877_at	ANXA2R	2.43	5.10	1.62E-06	0.000942					
202967_at	GSTA4	1.98	5.10	1.65E-06	0.000946	1.71	3.98	0.00014399	0.00040552	Yes
237680_at	---	1.68	5.09	1.70E-06	0.000963					
227592_at	ALDH16A1	1.41	5.09	1.72E-06	0.000968					

203349_s_at	ETV5	3.59	5.07	1.84E-06	0.001025	11.12	5.91	6.71E-08	2.99E-07	Yes
205448_s_at	MAP3K12	1.33	5.06	1.92E-06	0.001059	1.21	1.98	0.0508573	0.0806702	No
242598_at	---	3.04	5.02	2.29E-06	0.001249					
225752_at	NIPA1	2.68	5.01	2.34E-06	0.001263					
210174_at	NR5A2	1.95	5.00	2.45E-06	0.001308	-1.05	-0.35	0.72471679	0.77527842	No
228855_at	NUDT7	1.85	4.98	2.64E-06	0.001399					
234994_at	TMEM200A	2.15	4.98	2.70E-06	0.001415					
212265_at	QKI	-2.92	-4.97	2.79E-06	0.001447	-6.96	-3.80	0.00027296	0.00071071	Yes
235291_s_at	FLJ32255	2.10	4.95	3.03E-06	0.001556					
206335_at	GALNS	1.51	4.91	3.65E-06	0.001857	-1.28	-2.30	0.02372081	0.04091839	No
211685_s_at	NCALD	1.48	4.90	3.69E-06	0.001863	1.43	2.07	0.04098435	0.06576558	No
242414_at	QPRT	1.79	4.89	3.94E-06	0.001953					
209870_s_at	APBA2	1.65	4.89	3.94E-06	0.001953	1.18	0.91	0.36723531	0.42232061	No
226140_s_at	OTUD1	3.05	4.87	4.15E-06	0.00204					
205402_x_at	PRSS2	3.68	4.86	4.39E-06	0.002138	4.81	7.04	4.49E-10	2.39E-09	Yes
203836_s_at	MAP3K5	2.05	4.85	4.63E-06	0.002232	-1.20	-1.24	0.21879313	0.27448593	No
205349_at	GNA15	-2.90	-4.82	5.19E-06	0.002482	-1.06	-0.22	0.82327826	0.85422857	No
242461_at	---	1.37	4.81	5.46E-06	0.002588					
235173_at	MBNL1-AS1	1.34	4.79	5.81E-06	0.002719					
46665_at	SEMA4C	1.66	4.79	5.84E-06	0.002719	1.26	1.81	0.07439059	0.11038603	No
221696_s_at	STYK1	1.26	4.78	6.08E-06	0.002807	1.08	1.41	0.16215605	0.20913585	No
218409_s_at	DNAJC1	2.23	4.77	6.29E-06	0.002881	3.58	6.26	1.48E-08	7.04E-08	Yes
216048_s_at	RHOBTB3	1.69	4.74	7.11E-06	0.003229	-1.22	-1.11	0.27052346	0.32462815	No
214691_x_at	FAM63B	1.60	4.74	7.22E-06	0.003253	2.63	7.29	1.41E-10	8.15E-10	Yes
215164_at	---	1.91	4.73	7.42E-06	0.003317	1.68	7.60	3.34E-11	2.30E-10	Yes
205518_s_at	CMAHP	-4.24	-4.72	7.71E-06	0.003416	-3.01	-2.70	0.00837843	0.01583868	Yes
240570_at	INADL	1.33	4.70	8.33E-06	0.003661					
210033_s_at	SPAG6	2.50	4.69	8.74E-06	0.003794	4.29	4.69	1.02E-05	3.70E-05	Yes
213428_s_at	COL6A1	2.03	4.69	8.77E-06	0.003794	1.54	2.97	0.00390719	0.00804764	Yes
200984_s_at	CD59	2.24	4.67	9.39E-06	0.004003	3.19	4.45	2.60E-05	8.76E-05	Yes
236395_at	---	4.44	4.67	9.40E-06	0.004003					
244230_at	---	1.35	4.67	9.57E-06	0.004045					
239578_at	---	1.57	4.66	9.75E-06	0.004088					
212263_at	QKI	-4.40	-4.66	1.00E-05	0.004165	-2.58	-3.07	0.00288135	0.0062129	Yes
225710_at	GNB4	-3.97	-4.62	1.17E-05	0.004817					
204981_at	SLC22A18	1.40	4.60	1.25E-05	0.00512	1.31	1.90	0.06132887	0.09300423	No
229393_at	L3MBTL3	2.62	4.59	1.32E-05	0.005376					
226764_at	ZNF827	2.53	4.56	1.46E-05	0.005889					
206746_at	BFSP1	1.17	4.56	1.48E-05	0.005935	1.22	3.04	0.00312024	0.00662451	Yes
228221_at	SLC44A3	1.82	4.55	1.51E-05	0.005993					
209647_s_at	SOCS5	-1.67	-4.53	1.66E-05	0.006561	-1.23	-1.03	0.30420205	0.35576173	No
225639_at	SKAP2	4.31	4.51	1.76E-05	0.00683					
233280_at	---	1.53	4.51	1.77E-05	0.00683					
243618_s_at	ZNF827	2.40	4.51	1.78E-05	0.00683					
234107_s_at	DTD1	2.44	4.51	1.78E-05	0.00683					
213169_at	SEMA5A	1.44	4.49	1.91E-05	0.007238	1.07	0.69	0.49445545	0.55028107	No
223836_at	FGFBP2	2.47	4.49	1.91E-05	0.007238					
213693_s_at	MUC1	1.37	4.48	1.98E-05	0.007457	1.24	1.62	0.10796306	0.14898903	No
204675_at	SRD5A1	2.02	4.46	2.19E-05	0.008126	1.15	0.89	0.37465328	0.42729051	No
210032_s_at	SPAG6	1.61	4.46	2.19E-05	0.008126	1.51	2.95	0.00414736	0.00841669	Yes
240180_at	---	1.81	4.45	2.24E-05	0.008246					
242172_at	---	2.53	4.44	2.29E-05	0.008378					
209200_at	MEF2C	2.71	4.43	2.39E-05	0.008674	6.84	3.97	0.00014868	0.00041035	Yes
218380_at	LOC728392	2.38	4.43	2.41E-05	0.00868	2.47	4.25	5.36E-05	0.00016429	Yes
243617_at	ZNF827	2.11	4.43	2.42E-05	0.00868					
210542_s_at	SLCO3A1	1.39	4.43	2.47E-05	0.008766	1.20	2.23	0.02819639	0.04803829	Yes
229253_at	THEM4	1.91	4.42	2.48E-05	0.008766					
243492_at	THEM4	1.39	4.41	2.62E-05	0.00921					
232629_at	PROK2	2.36	4.41	2.64E-05	0.009211					
213644_at	CEP112	1.40	4.39	2.86E-05	0.009849	1.21	2.54	0.01274835	0.02314832	Yes
222111_at	FAM63B	2.63	4.39	2.87E-05	0.009849	3.98	7.18	2.33E-10	1.28E-09	Yes
214752_x_at	FLNA	1.59	4.39	2.88E-05	0.009849	1.42	1.57	0.11893431	0.15934889	No
1560018_at	ARPP21	1.77	4.38	2.89E-05	0.009849					

215307_at	ZNF529	-2.52	-4.38	2.96E-05	0.01002	-1.95	-2.63	0.01017707	0.01897885	Yes
219225_at	PGBD5	1.46	4.37	3.01E-05	0.010128	1.21	1.72	0.08920732	0.12823552	No
211056_s_at	SRD5A1	1.42	4.37	3.04E-05	0.010161	1.20	1.94	0.05603917	0.08592673	No
206361_at	PTGDR2	1.39	4.35	3.28E-05	0.010892	2.29	3.94	0.00016703	0.00045196	Yes
241936_x_at	---	1.44	4.35	3.36E-05	0.011097					
235957_at	GRIP1	1.74	4.32	3.76E-05	0.012276					
230102_at	ETV5	2.00	4.31	3.78E-05	0.012276					
200983_x_at	CD59	2.00	4.31	3.78E-05	0.012276	2.59	2.98	0.00378056	0.00790481	Yes
211555_s_at	GUCY1B3	1.60	4.31	3.85E-05	0.012423	5.38	4.67	1.09E-05	3.85E-05	Yes
1556842_at	LOC286087	2.12	4.30	4.04E-05	0.012898					
229636_at	---	1.26	4.30	4.05E-05	0.012898					
205578_at	ROR2	1.25	4.29	4.12E-05	0.013063	-1.03	-0.62	0.53824844	0.59422628	No
227399_at	VGLL3	1.92	4.28	4.30E-05	0.013557					
207463_x_at	PRSS2 /// PRSS3	2.08	4.26	4.64E-05	0.014549	2.78	4.54	1.82E-05	6.29E-05	Yes
219746_at	DPF3	1.54	4.26	4.68E-05	0.014582	1.92	7.72	1.95E-11	1.41E-10	Yes
202970_at	DYRK2	1.64	4.26	4.74E-05	0.014667	1.06	0.80	0.42479538	0.48050625	No
202255_s_at	SIPA1L1	1.46	4.25	4.81E-05	0.014807	1.01	0.07	0.9426252	0.9608047	No
204362_at	SKAP2	3.26	4.24	5.00E-05	0.015302	3.03	3.24	0.00170119	0.00384859	Yes
216470_x_at	PRSS2	2.34	4.22	5.42E-05	0.016506	3.26	6.82	1.21E-09	6.18E-09	Yes
241782_at	NEBL	1.30	4.21	5.55E-05	0.01677					
220102_at	FOXL2	1.84	4.21	5.57E-05	0.01677	-1.11	-1.52	0.13163138	0.17300124	No
227798_at	SMAD1	3.92	4.21	5.61E-05	0.016804					
236901_at	---	1.28	4.19	5.98E-05	0.017806					
1560662_s_at	WHAMMP2 /// WHAMMP3	2.10	4.19	6.11E-05	0.018091					
232051_at	CCDC102A	1.27	4.19	6.14E-05	0.018091					
209581_at	PLA2G16	1.52	4.18	6.17E-05	0.018091	1.16	1.12	0.26540928	0.32128492	No
203348_s_at	ETV5	1.75	4.18	6.31E-05	0.018404	3.14	4.87	5.00E-06	1.91E-05	Yes
225280_x_at	ARSD	1.35	4.17	6.50E-05	0.018831					
204044_at	QPRT	1.67	4.16	6.66E-05	0.019138	1.45	2.16	0.0332658	0.0553094	No
210959_s_at	SRD5A1	1.41	4.16	6.70E-05	0.019138	-1.11	-1.05	0.29548575	0.34852165	No
211382_s_at	TACC2	1.44	4.16	6.71E-05	0.019138	1.19	1.79	0.07653169	0.11235504	No
226981_at	KMT2A	-1.97	-4.16	6.82E-05	0.019368					
213737_x_at	GOLGA8I /// GOLGA8O	3.26	4.15	6.93E-05	0.019569	5.41	2.87	0.00521624	0.01028344	Yes
228748_at	CD59	1.39	4.15	7.04E-05	0.019771					
208127_s_at	SOCS5	-2.33	-4.14	7.37E-05	0.020602	-1.32	-1.28	0.20297666	0.25697962	No
232597_x_at	SCAF11	1.56	4.13	7.54E-05	0.020978					
234423_x_at	LOC100996255	1.50	4.12	7.79E-05	0.02154					
1560758_at	---	1.31	4.12	7.97E-05	0.021948					
238498_at	OTTHUMG00000175943 /// RP3-406A7.7	1.57	4.11	8.12E-05	0.022245					
209648_x_at	SOCS5	-2.49	-4.11	8.19E-05	0.022314	-1.24	-1.13	0.26286341	0.32101903	No
239296_at	---	1.60	4.11	8.30E-05	0.022498					
231902_at	ZNF827	1.57	4.10	8.41E-05	0.022699					
205229_s_at	COCH	1.74	4.10	8.51E-05	0.022852	1.90	3.20	0.00189727	0.00422297	Yes
201997_s_at	SPEN	-1.95	-4.09	8.66E-05	0.023133	-1.27	-1.58	0.11823278	0.15934889	No
200985_s_at	CD59	2.88	4.08	8.95E-05	0.023788	5.43	4.02	0.00012656	0.0003716	Yes
1560512_at	---	1.45	4.08	9.08E-05	0.024026					
226971_at	CCDC136	1.35	4.07	9.47E-05	0.024927					
220488_s_at	BCAS3	1.37	4.07	9.58E-05	0.025092	1.10	0.97	0.33494499	0.3884236	No
232298_at	MBNL1-AS1	1.17	4.04	0.0001036	0.027022					
226873_at	FAM63B	2.88	4.04	0.0001055	0.027395					
227769_at	GPR27	2.29	4.03	0.0001081	0.027914					
216373_at	TAPT1	2.65	4.03	0.0001108	0.028483	1.13	2.83	0.00574261	0.01116169	Yes

235758_at	PNMA6A /// PNMA6B /// PNMA6C /// PNMA6D	1.47	4.03	0.0001113	0.028483					
202975_s_at	RHOBTB3	2.24	4.02	0.0001121	0.02854	-1.12	-0.53	0.59435625	0.65096161	No
1568589_at	---	1.51	4.02	0.0001149	0.029117					
1555963_x_at	B3GNT7	1.31	4.01	0.0001176	0.029684					
205572_at	ANGPT2	1.55	4.00	0.0001219	0.03063	-1.01	-0.06	0.95384235	0.9608047	No
229461_x_at	NEGR1	3.32	3.99	0.0001242	0.031058					
236008_at	LOC100128909	1.35	3.98	0.0001327	0.033016					
239842_x_at	---	1.36	3.97	0.0001355	0.033559					
205471_s_at	DACH1	2.08	3.97	0.0001368	0.033735	1.33	1.94	0.05540449	0.08590809	No
202908_at	WFS1	1.38	3.97	0.0001384	0.033918	2.18	5.03	2.67E-06	1.12E-05	Yes
1554703_at	ARHGEF10	1.56	3.96	0.0001388	0.033918					
242055_at	PSMG4	1.93	3.96	0.0001402	0.033924					
214930_at	SLITRK5	1.50	3.96	0.0001403	0.033924	1.13	1.71	0.09114866	0.12967542	No
238850_at	LINC00461 /// MIR9-2	2.29	3.96	0.000141	0.033924					
215802_at	---	1.58	3.96	0.0001413	0.033924	1.03	0.33	0.73953186	0.77904883	No
243049_at	---	1.45	3.95	0.0001437	0.034324					
202254_at	SIPA1L1	1.73	3.95	0.0001442	0.034324	1.08	0.31	0.75742054	0.79184874	No
214535_s_at	ADAMTS2	1.26	3.92	0.0001606	0.037956	1.40	3.53	0.00066598	0.00161236	Yes
214639_s_at	HOXA1	1.35	3.92	0.0001612	0.037956	-1.00	-0.02	0.98521935	0.98521935	No
228412_at	LOC643072	1.33	3.92	0.0001619	0.037956					
209572_s_at	EED	-2.02	-3.92	0.0001623	0.037956	-1.12	-0.50	0.61951817	0.67317723	No
240321_at	---	1.52	3.92	0.0001646	0.038303					
218831_s_at	FCGRT	1.55	3.92	0.0001651	0.038303	1.82	2.50	0.01425176	0.02554212	Yes
203870_at	USP46	1.70	3.91	0.0001662	0.038395	1.34	1.58	0.11861987	0.15934889	No
210304_at	PDE6B	1.29	3.90	0.000174	0.040008	1.09	1.22	0.22580132	0.28072596	No
221610_s_at	STAP2	-1.28	-3.89	0.0001792	0.041047	-1.20	-1.62	0.1078463	0.14898903	No
213421_x_at	PRSS2 /// PRSS3	2.00	3.89	0.0001819	0.041443	2.29	4.06	0.0001074	0.0003222	Yes
219860_at	LY6G5C	1.25	3.89	0.0001825	0.041443	1.17	1.36	0.17609268	0.22500731	No
225949_at	NRBP2	1.75	3.88	0.0001864	0.042165					
215197_at	---	1.31	3.88	0.0001876	0.04225	1.04	0.46	0.64404887	0.69436519	No
237058_x_at	SLC6A13	1.93	3.88	0.0001898	0.04256					
225286_at	ARSD	1.39	3.88	0.0001908	0.042614					
244480_at	---	1.33	3.87	0.0001938	0.043123					
226415_at	VAT1L	1.47	3.87	0.0001961	0.043457					
220030_at	STYK1	1.28	3.86	0.0001998	0.044085	1.10	1.53	0.12968271	0.17207898	No
226311_at	ADAMTS2	1.38	3.86	0.000201	0.044174					
231035_s_at	OTUD1	1.54	3.86	0.0002046	0.044785					
1555842_at	CYTH2	1.53	3.85	0.0002118	0.046085					
236632_at	HHIP-AS1	2.20	3.84	0.0002138	0.046085					
211707_s_at	IQCB1	-1.55	-3.84	0.0002138	0.046085	-1.34	-2.09	0.03982135	0.06465114	No
204671_s_at	ANKRD6	1.61	3.84	0.0002139	0.046085	1.18	1.07	0.28724692	0.34172478	No
1562194_at	---	1.81	3.84	0.0002157	0.046295					
1566903_at	---	1.31	3.84	0.0002169	0.046357					
205488_at	GZMA	3.78	3.84	0.0002202	0.046892	-1.06	-0.15	0.88409618	0.91048712	No
205826_at	MYOM2	2.84	3.82	0.0002298	0.048729	2.91	2.82	0.00598536	0.01147194	Yes
218376_s_at	MICAL1	2.13	3.82	0.0002363	0.049926	1.95	2.60	0.01092152	0.02009559	Yes

MLLT10-R vs Others

Probe Set ID	Gene Symbol	COG Data				Soulier's Data				Validated
		old Chag	t	p-value	FDR	old Chag	t	p-value	FDR	
213844_at	HOXA5	25.20	9.58	8.52E-16	4.64E-11	18.12	7.68	2.17E-11	1.30E-10	Yes
209763_at	CHRDL1	7.06	9.13	8.54E-15	2.33E-10	5.23	5.48	4.07E-07	7.32E-07	Yes
235521_at	HOXA3	8.55	8.71	6.86E-14	1.25E-09					
1559477_s_at	MEIS1	8.04	7.22	1.08E-10	1.22E-06					
235753_at	HOXA7	2.98	7.21	1.12E-10	1.22E-06					
214639_s_at	HOXA1	1.93	7.15	1.47E-10	1.33E-06	1.44	2.40	0.0183621	0.02065736	Yes
204069_at	MEIS1	9.85	7.11	1.79E-10	1.39E-06	4.23	7.11	3.07E-10	1.10E-09	Yes

206289_at	HOXA4	1.97	6.90	5.01E-10	3.41E-06	1.91	7.55	4.01E-11	1.80E-10	Yes
1559266_s_at	SKIDA1	3.38	6.78	8.71E-10	5.27E-06					
209905_at	HOXA10-HOXA9	25.73	6.47	3.65E-09	1.99E-05	20.60	7.79	1.35E-11	1.22E-10	Yes
214651_s_at	HOXA10-HOXA9	33.07	6.43	4.53E-09	2.25E-05	37.81	6.51	4.62E-09	1.21E-08	Yes
242172_at	---	4.93	6.38	5.78E-09	2.62E-05					
206847_s_at	HOXA7	2.93	6.16	1.54E-08	6.45E-05	1.87	7.82	1.14E-11	1.22E-10	Yes
243003_at	---	3.53	6.04	2.63E-08	0.000102					
210033_s_at	SPAG6	4.04	5.97	3.67E-08	0.000131	4.30	5.60	2.44E-07	4.88E-07	Yes
227949_at	PHACTR3	2.39	5.96	3.92E-08	0.000131					
205600_x_at	HOXB5	1.46	5.95	4.10E-08	0.000131	1.09	1.07	0.28599267	0.30281577	No
210032_s_at	SPAG6	2.13	5.89	5.35E-08	0.000162	1.68	4.39	3.22E-05	4.82E-05	Yes
228116_at	---	4.12	5.81	7.40E-08	0.000212					
1553808_a_at	NKX2-3	1.81	5.63	1.70E-07	0.000463					
242881_x_at	LOC100506303 /	7.94	5.59	1.98E-07	0.000513					
237600_at	---	2.84	5.56	2.29E-07	0.000567					
242216_at	---	7.79	5.39	4.84E-07	0.001146					
235149_at	PGM2L1	1.48	5.31	6.78E-07	0.00154					
232587_at	EML4	2.31	5.21	1.02E-06	0.002213					
227195_at	ZNF503	4.35	5.13	1.44E-06	0.003021					
231374_at	---	1.99	5.10	1.66E-06	0.003352					
202265_at	BMI1 /// COMMI	4.14	4.89	3.95E-06	0.00751	4.42	5.89	7.02E-08	1.58E-07	Yes
208557_at	HOXA6	1.86	4.88	4.00E-06	0.00751	1.33	3.32	0.00133467	0.00160161	Yes
213150_at	HOXA10	6.47	4.87	4.17E-06	0.007582	7.62	5.08	2.10E-06	3.44E-06	Yes
1555923_a_at	C10orf114	1.58	4.73	7.35E-06	0.012915					
205408_at	MLLT10	1.85	4.71	8.18E-06	0.013927	1.21	1.03	0.30410623	0.30410623	No
219988_s_at	RNF220	1.77	4.66	1.00E-05	0.016558	1.80	6.51	4.71E-09	1.21E-08	Yes
229667_s_at	HOXB8	1.51	4.54	1.61E-05	0.025785					
1559265_at	SKIDA1	1.40	4.52	1.70E-05	0.026507					
225992_at	MLLT10	2.55	4.48	1.96E-05	0.029703					
213147_at	HOXA10	2.31	4.47	2.04E-05	0.030068	2.84	3.60	0.00052405	0.00067378	Yes
228708_at	RAB27B	4.21	4.45	2.24E-05	0.032192					
1569448_at	PGM2L1	2.00	4.36	3.22E-05	0.045032					
212265_at	QKI	-3.07	-4.34	3.41E-05	0.046454	-6.08	-4.07	0.00010424	0.00014434	Yes

**MLLT10-R vs KMT2A-R**										
Probe Set ID	Gene Symbol	COG Data				Soulier's Data				Validated
		Fold Chag	t	p-value	FDR	Fold Chag	t	p-value	FDR	
203216_s_at	MYO6	-11.70	-8.01	2.21E-12	1.20E-07	-19.97	-31.65	1.47E-08	2.80E-07	Yes
236859_at	RUNX2	-4.85	-7.72	9.21E-12	2.51E-07					
236858_s_at	RUNX2	-3.42	-7.59	1.73E-11	3.15E-07					
203215_s_at	MYO6	-4.93	-7.14	1.52E-10	2.08E-06	-2.00	-4.60	0.00276683	0.00438081	Yes
226939_at	CPEB2	-10.63	-5.97	3.64E-08	0.000397					
240927_at	---	-3.24	-5.92	4.67E-08	0.000424					
240468_at	---	-4.26	-5.80	7.89E-08	0.000615					
210480_s_at	MYO6	-1.90	-5.31	6.74E-07	0.004406	-4.36	-10.44	2.15E-05	6.82E-05	Yes
213908_at	WHAMMP2 /// V	-4.69	-5.29	7.27E-07	0.004406	-3.39	-6.62	0.00035703	0.00084794	Yes
209763_at	CHRDL1	4.00	5.26	8.22E-07	0.004482	3.66	5.42	0.00113568	0.00196162	Yes
228837_at	TCF4	-1.59	-5.17	1.23E-06	0.006089					
1557261_at	WHAMMP2 /// V	-2.41	-5.12	1.50E-06	0.006832					
244105_at	WHAMMP2 /// V	-1.50	-5.00	2.45E-06	0.010287					
214705_at	INADL	-2.52	-4.88	4.13E-06	0.015277	-1.00	-0.03	0.97913211	0.97913211	No
1553145_at	TAPT1-AS1	-2.10	-4.87	4.20E-06	0.015277					
243617_at	ZNF827	-3.31	-4.83	4.99E-06	0.016887					
212382_at	TCF4	-5.11	-4.82	5.27E-06	0.016887	-7.71	-16.43	1.14E-06	1.08E-05	Yes
232587_at	EML4	2.58	4.80	5.61E-06	0.016992					
226297_at	HIPK3	-3.13	-4.73	7.57E-06	0.020503					
213891_s_at	TCF4	-7.54	-4.72	7.77E-06	0.020503	-18.61	-15.23	1.88E-06	1.19E-05	Yes
214772_at	KIAA1549L	-1.76	-4.71	7.90E-06	0.020503	-1.96	-4.48	0.00317628	0.00464226	Yes
228116_at	---	4.08	4.69	8.68E-06	0.021503					
222146_s_at	TCF4	-4.44	-4.65	1.01E-05	0.023948	-11.95	-10.45	2.14E-05	6.82E-05	Yes
212387_at	TCF4	-5.04	-4.64	1.06E-05	0.023959	-11.50	-9.31	4.44E-05	0.00012049	Yes
243618_s_at	ZNF827	-3.74	-4.61	1.21E-05	0.026329					
235479_at	CPEB2	-2.32	-4.59	1.29E-05	0.026975					
242881_x_at	LOC100506303 /	8.08	4.58	1.34E-05	0.027027					

206506_s_at	SUPT3H	-2.59	-4.55	1.53E-05	0.029778	-4.27	-5.61	0.00093275	0.00177222	Yes
203753_at	TCF4	-5.44	-4.51	1.78E-05	0.033501	-5.85	-6.47	0.00040892	0.00086327	Yes
212386_at	TCF4	-4.56	-4.50	1.87E-05	0.033983	-24.56	-13.44	4.24E-06	2.01E-05	Yes
1553808_a_at	NKX2-3	1.79	4.47	2.09E-05	0.036713					
215163_at	---	-3.64	-4.44	2.30E-05	0.038004	-1.17	-0.76	0.47213886	0.52768461	No
210993_s_at	SMAD1	-5.57	-4.44	2.30E-05	0.038004	-4.49	-1.44	0.19560037	0.23227544	No
1556739_at	GOLGA8I	-1.66	-4.41	2.58E-05	0.041438					
209871_s_at	APBA2	-2.72	-4.40	2.74E-05	0.041559	1.10	0.20	0.84444383	0.89135738	No
204304_s_at	PROM1	-20.73	-4.40	2.74E-05	0.041559	-37.53	-3.90	0.00637213	0.00864789	Yes
231374_at	---	2.07	4.38	2.94E-05	0.043381					
202263_at	CYB5R1	-1.77	-4.36	3.17E-05	0.045502	-1.40	-1.97	0.09144352	0.11582846	No
