# Supplementary material for: Dysregulated transcriptional networks in KMT2A- and MLLT10-rearranged T-ALL
Source: Biomark Res. 2018 Aug 23;6:27. doi: 10.1186/s40364-018-0141-z (PMC6107954; doi:10.1186/s40364-018-0141-z)
Supplement: Supplementary file 4 — Table S3. Validation of gene expression for MLLT10-R. (PDF 243 kb) [file 40364_2018_141_MOESM4_ESM.pdf]

Supplementary Table S3. Gene expression profiling for MLLT10-R in a molecular interrogation cohort of 100 T-ALL drawn from COG AALL0434 study and the validation cohorts of Soulier et al. (2005) and Dik et al. (2005). Differentially expressed genes in MLL-R T-ALL vs Others ("Others" reflecting cases that do not have either KMT2A-R or MLLT10-R.), MLLT10-R vs Others, MLLT10-R10 vs. KMT2A-R

MLLT10-R vs Others

Probe Set ID	Gene Symbol	COG Data				Soulier's Data				
		Fold Chage	t	p-value	FDR	Fold Chage	t	p-value	FDR	Validated
213844_at	HOXA5	25.20	9.58	8.52E-16	4.64E-11	18.12	7.68	2.17E-11	1.30E-10	Yes
209763_at	CHRDL1	7.06	9.13	8.54E-15	2.33E-10	5.23	5.48	4.07E-07	7.32E-07	Yes
235521_at	HOXA3	8.55	8.71	6.86E-14	1.25E-09					
1559477_s_at	MEIS1	8.04	7.22	1.08E-10	1.22E-06					
235753_at	HOXA7	2.98	7.21	1.12E-10	1.22E-06					
214639_s_at	HOXA1	1.93	7.15	1.47E-10	1.33E-06	1.44	2.40	0.0183621	0.0206574	Yes
204069_at	MEIS1	9.85	7.11	1.79E-10	1.39E-06	4.23	7.11	3.07E-10	1.10E-09	Yes
206289_at	HOXA4	1.97	6.90	5.01E-10	3.41E-06	1.91	7.55	4.01E-11	1.80E-10	Yes
1559266_s_at	SKIDA1	3.38	6.78	8.71E-10	5.27E-06					
209905_at	HOXA10-HOXA9 /// HOXA9 /// MIR196B	25.73	6.47	3.65E-09	1.99E-05	20.60	7.79	1.35E-11	1.22E-10	Yes
214651_s_at	HOXA10-HOXA9 /// HOXA9 /// MIR196B	33.07	6.43	4.53E-09	2.25E-05	37.81	6.51	4.62E-09	1.21E-08	Yes
242172_at	---	4.93	6.38	5.78E-09	2.62E-05					
206847_s_at	HOXA7	2.93	6.16	1.54E-08	6.45E-05	1.87	7.82	1.14E-11	1.22E-10	Yes
243003_at	---	3.53	6.04	2.63E-08	0.000102291					
210033_s_at	SPAG6	4.04	5.97	3.67E-08	0.000131302	4.30	5.60	2.44E-07	4.88E-07	Yes
227949_at	PHACTR3	2.39	5.96	3.92E-08	0.000131302					
205600_x_at	HOXB5	1.46	5.95	4.10E-08	0.000131302	1.09	1.07	0.2859927	0.3028158	No
210032_s_at	SPAG6	2.13	5.89	5.35E-08	0.000161996	1.68	4.39	3.22E-05	4.82E-05	Yes
228116_at	---	4.12	5.81	7.40E-08	0.000212321					
1553808_a_at	NKX2-3	1.81	5.63	1.70E-07	0.000462977					
242881_x_at	LOC100506303 /// LOC100653149 /// LOC101060483 /// LOC400879 /// LOC440157	7.94	5.59	1.98E-07	0.000513061					
237600_at	---	2.84	5.56	2.29E-07	0.000566645					
242216_at	---	7.79	5.39	4.84E-07	0.001146214					
235149_at	PGM2L1	1.48	5.31	6.78E-07	0.001539902					
232587_at	EML4	2.31	5.21	1.02E-06	0.002213083					
227195_at	ZNF503	4.35	5.13	1.44E-06	0.003021053					
231374_at	---	1.99	5.10	1.66E-06	0.00335235					
202265_at	BMI1 /// COMMD3-	4.14	4.89	3.95E-06	0.007510162	4.42	5.89	7.02E-08	1.58E-07	Yes
208557_at	HOXA6	1.86	4.88	4.00E-06	0.007510162	1.33	3.32	0.0013347	0.0016016	Yes
213150_at	HOXA10	6.47	4.87	4.17E-06	0.007582198	7.62	5.08	2.10E-06	3.44E-06	Yes
1555923_a_at	C10orf114	1.58	4.73	7.35E-06	0.012914977					
205408_at	MLLT10	1.85	4.71	8.18E-06	0.013927419	1.21	1.03	0.3041062	0.3041062	No
219988_s_at	RNF220	1.77	4.66	1.00E-05	0.016557975	1.80	6.51	4.71E-09	1.21E-08	Yes
229667_s_at	HOXB8	1.51	4.54	1.61E-05	0.025784551					
1559265_at	SKIDA1	1.40	4.52	1.70E-05	0.026507439					
225992_at	MLLT10	2.55	4.48	1.96E-05	0.029702992					
213147_at	HOXA10	2.31	4.47	2.04E-05	0.030068449	2.84	3.60	0.000524	0.0006738	Yes
228708_at	RAB27B	4.21	4.45	2.24E-05	0.032191537					
1569448_at	PGM2L1	2.00	4.36	3.22E-05	0.045032336					
212265_at	QKI	-3.07	-4.34	3.41E-05	0.046453564	-6.08	-4.07	0.0001042	0.0001443	Yes
**Dik's Data**										
						Fold Chage	t	p-value	FDR	Validated
213844_at	HOXA5	25.20	9.58	8.52E-16	4.64E-11	3.91	3.69	0.001178	0.007536	Yes

209763_at	CHRDL1	7.06	9.13	8.54E-15	2.33E-10	-1.00	0.00	0.999058	0.999058	No
235521_at	HOXA3	8.55	8.71	6.86E-14	1.25E-09					
1559477_s_at	MEIS1	8.04	7.22	1.08E-10	1.22E-06					
235753_at	HOXA7	2.98	7.21	1.12E-10	1.22E-06					
214639_s_at	HOXA1	1.93	7.15	1.47E-10	1.33E-06	1.09	0.52	0.607439	0.683368	No
204069_at	MEIS1	9.85	7.11	1.79E-10	1.39E-06	1.71	1.91	0.068446	0.115356	No
206289_at	HOXA4	1.97	6.90	5.01E-10	3.41E-06	1.11	1.22	0.233930	0.350894	No
1559266_s_at	SKIDA1	3.38	6.78	8.71E-10	5.27E-06					
209905_at	HOXA10-HOXA9 /// HOXA9 /// MIR196B	25.73	6.47	3.65E-09	1.99E-05	3.90	4.81	0.000071	0.001270	Yes
214651_s_at	HOXA10-HOXA9 /// HOXA9 /// MIR196B	33.07	6.43	4.53E-09	2.25E-05	7.27	3.66	0.001256	0.007536	Yes
242172_at	---	4.93	6.38	5.78E-09	2.62E-05					
206847_s_at	HOXA7	2.93	6.16	1.54E-08	6.45E-05	1.52	2.63	0.014791	0.038034	Yes
243003_at	---	3.53	6.04	2.63E-08	0.000102291					
210033_s_at	SPAG6	4.04	5.97	3.67E-08	0.000131302	2.74	2.49	0.020343	0.045772	Yes
227949_at	PHACTR3	2.39	5.96	3.92E-08	0.000131302					
205600_x_at	HOXB5	1.46	5.95	4.10E-08	0.000131302	1.12	0.74	0.464982	0.597835	No
210032_s_at	SPAG6	2.13	5.89	5.35E-08	0.000161996	1.23	1.13	0.270139	0.374038	No
228116_at	---	4.12	5.81	7.40E-08	0.000212321	3.03	3.53	0.001727	0.007772	Yes
1553808_a_at	NKX2-3	1.81	5.63	1.70E-07	0.000462977	1.26	1.89	0.070495	0.115356	No
242881_x_at	LOC100506303 /// LOC100653149 /// LOC101060483 /// LOC400879 /// LOC440157	7.94	5.59	1.98E-07	0.000513061					
237600_at	---	2.84	5.56	2.29E-07	0.000566645					
242216_at	---	7.79	5.39	4.84E-07	0.001146214					
235149_at	PGM2L1	1.48	5.31	6.78E-07	0.001539902					
232587_at	EML4	2.31	5.21	1.02E-06	0.002213083					
227195_at	ZNF503	4.35	5.13	1.44E-06	0.003021053					
231374_at	---	1.99	5.10	1.66E-06	0.00335235					
202265_at	BMI1 /// COMMD3-	4.14	4.89	3.95E-06	0.007510162					
208557_at	HOXA6	1.86	4.88	4.00E-06	0.007510162					
213150_at	HOXA10	6.47	4.87	4.17E-06	0.007582198	7.62	5.08	2.10E-06	3.44E-06	Yes
1555923_a_at	C10orf114	1.58	4.73	7.35E-06	0.012914977					
205408_at	MLLT10	1.85	4.71	8.18E-06	0.013927419	1.21	1.03	0.3041062	0.3041062	No
219988_s_at	RNF220	1.77	4.66	1.00E-05	0.016557975	1.80	6.51	4.71E-09	1.21E-08	Yes
229667_s_at	HOXB8	1.51	4.54	1.61E-05	0.025784551					
1559265_at	SKIDA1	1.40	4.52	1.70E-05	0.026507439					
225992_at	MLLT10	2.55	4.48	1.96E-05	0.029702992					
213147_at	HOXA10	2.31	4.47	2.04E-05	0.030068449	2.84	3.60	0.000524	0.0006738	Yes
228708_at	RAB27B	4.21	4.45	2.24E-05	0.032191537					
1569448_at	PGM2L1	2.00	4.36	3.22E-05	0.045032336					
212265_at	QKI	-3.07	-4.34	3.41E-05	0.046453564	-6.08	-4.07	0.0001042	0.0001443	Yes
