# Supplementary material for: Dysregulated transcriptional networks in KMT2A- and MLLT10-rearranged T-ALL
Source: Biomark Res. 2018 Aug 23;6:27. doi: 10.1186/s40364-018-0141-z (PMC6107954; doi:10.1186/s40364-018-0141-z)
Supplement: Supplementary file 5 — Table S4. GSEA for gene ontology. (PDF 265 kb) [file 40364_2018_141_MOESM5_ESM.pdf]

Supplementary Table S4. GSEA analyses for gene ontology in T-ALL harboring KMT2A-R or MLLT10-R rearrangements compared to Others.

Name	SIZE	KMT2A-R vs Others (1)		MLLT10-R vs Others (2)		MLLT10-R vs KMT2A-R (3)		flag
		NES	FDR	NES	FDR	NES	FDR	
GO_DNA_PACKAGING_COMPLEX	82	-3.532	0.000	-3.761	0.000			12
GO_NUCLEAR_NUCLEOSOME	33	-3.438	0.000	-3.627	0.000			12
GO_INNATE_IMMUNE_RESPONSE_IN_MUCOSA	17	-2.814	0.000	-2.830	0.000			12
GO_CYTOSOLIC_RIBOSOME	94	-2.786	0.000	-1.775	0.029			12
GO_ESTABLISHMENT_OF_PROTEIN_LOCALIZATION_TO_ENDOPLASMIC_RETICULUM	97	-2.764	0.000					1
GO_NUCLEAR_TRANSCRIBED_MRNA_CATABOLIC_PROCESS_NONSENSE_MEDIATED_DECAY	105	-2.718	0.000					1
GO_ORGAN_OR_TISSUE_SPECIFIC_IMMUNE_RESPONSE	26	-2.663	0.000	-2.603	0.000			12
GO_CYTOSOLIC_LARGE_RIBOSOMAL_SUBUNIT	50	-2.648	0.000					1
GO_PROTEIN_DNA_COMPLEX	140	-2.611	0.000	-3.023	0.000			12
GO_DNA_REPLICATION_DEPENDENT_NUCLEOSOME_ORGANIZATION	26	-2.598	0.000	-2.899	0.000			12
GO_DNA_REPLICATION_DEPENDENT_NUCLEOSOME_ASSEMBLY	26	-2.587	0.000	-2.862	0.000			12
GO_DNA_PACKAGING	152	-2.516	0.000	-2.989	0.000			12
GO_TRANSLATIONAL_INITIATION	117	-2.507	0.000	-1.739	0.036			12
GO_CHROMATIN_SILENCING	69	-2.490	0.000	-2.550	0.000			12
GO_RIBOSOMAL_SUBUNIT	144	-2.489	0.000	-2.405	0.000			12
GO_RRNA_METABOLIC_PROCESS	196	-2.470	0.000	-2.014	0.008			12
GO_LARGE_RIBOSOMAL_SUBUNIT	82	-2.461	0.000	-2.123	0.004			12
GO_CHROMATIN_ASSEMBLY_OR_DISASSEMBLY	142	-2.446	0.000	-3.150	0.000			12
GO_PROTEIN_LOCALIZATION_TO_ENDOPLASMIC_RETICULUM	114	-2.444	0.000					1
GO_DNA_CONFORMATION_CHANGE	224	-2.434	0.000	-2.545	0.000			12
GO_CYTOSOLIC_SMALL_RIBOSOMAL_SUBUNIT	38	-2.412	0.000	-1.947	0.012			12
GO_CATALYTIC_STEP_2_SPLICEOSOME	66	-2.391	0.000					1
GO_CHROMATIN_SILENCING_AT_RDNA	26	-2.373	0.000	-2.839	0.000			12
GO_MULTI_ORGANISM_METABOLIC_PROCESS	124	-2.371	0.000					1
GO_MATURATION_OF_SSU_RRNA	32	-2.361	0.000	-1.973	0.010			12
GO_NEGATIVE_REGULATION_OF_GENE_EXPRESSION_EPIGENETIC	85	-2.338	0.001	-2.159	0.003			12
GO_PROTEIN_DNA_COMPLEX_SUBUNIT_ORGANIZATION	190	-2.287	0.001	-2.451	0.000			12
GO_PRECATALYTIC_SPLICEOSOME	18	-2.219	0.002					1
GO_U12_TYPE_SPLICEOSOMAL_COMPLEX	22	-2.147	0.004					1
GO_RIBOSOME_BIOGENESIS	240	-2.117	0.005	-1.900	0.016			12
GO_STRUCTURAL_CONSTITUENT_OF_RIBOSOME	191	-2.109	0.005	-2.040	0.007			12
GO_SPLICEOSOMAL_COMPLEX	128	-2.088	0.006					1
GO_ERROR_PRONE_TRANSLESION_SYNTHESIS	18	-2.080	0.006	-2.202	0.002			12
GO_U2_SNRNP	19	-2.078	0.006					1
GO_PROTEIN_HETEROTETRAMERIZATION	32	-2.070	0.006	-2.585	0.000			12
GO_RIBOSOME_ASSEMBLY	41	-2.022	0.009	-1.685	0.047			12
GO_PROTEASOME_ACCESSORY_COMPLEX	23	-2.014	0.009					1
GO_RIBOSOMAL_SMALL_SUBUNIT_BIOGENESIS	46	-1.982	0.011	-1.878	0.018			12
GO_RIBOSOME	199	-1.959	0.014	-2.055	0.006			12

GO_RIBONUCLEOPROTEIN_COMPLEX_LOCALIZATION	94	-1.959	0.014					1
GO_PROTEIN_TARGETING_TO_MEMBRANE	144	-1.952	0.014					1
GO_CYTOSOLIC_PART	181	-1.948	0.014	-1.698	0.044			12
GO_TELOMERE_ORGANIZATION	86	-1.923	0.017	-2.995	0.000			12
GO_MATURATION_OF_SSU_RRNA_FROM_TRICISTRONIC_RRNA_TRANSCRIPT_SSU_RRNA_5_8S_RRNA_LSU_RRNA_	24	-1.901	0.019	-1.771	0.030			12
GO_SMALL_RIBOSOMAL_SUBUNIT	62	-1.885	0.022	-2.408	0.000			12
GO_NUCLEAR_CHROMATIN	254	-1.885	0.021	-2.341	0.001			12
GO_POSITIVE_REGULATION_OF_GENE_EXPRESSION_EPIGENETIC	63	-1.851	0.027	-2.139	0.004			12
GO_ER_NUCLEUS_SIGNALING_PATHWAY	31	-1.830	0.032					1
GO_DEFENSE_RESPONSE_TO_GRAM_POSITIVE_BACTERIUM	63	-1.825	0.032	-1.894	0.017			12
GO_RIBOSOMAL_LARGE_SUBUNIT_ASSEMBLY	18	-1.821	0.033					1
GO_NUCLEAR_EXPORT	117	-1.805	0.036					1
GO_SMALL_NUCLEAR_RIBONUCLEOPROTEIN_COMPLEX	53	-1.801	0.036					1
GO_RNA_SPLICING_VIA_TRANSESTERIFICATION_REACTIONS	197	-1.795	0.037					1
GO_CYTOPLASMIC_TRANSLATION	27	-1.794	0.037	-2.004	0.008			12
GO_RRNA_BINDING	48	-1.787	0.038					1
GO_UTERUS_DEVELOPMENT	17	2.110	0.002					1
GO_FIBRIL_ORGANIZATION	19	2.066	0.004					1
GO_EMBRYONIC_SKELETAL_SYSTEM_MORPHOGENESIS	88	1.952	0.050	2.352	0.000			12
GO_MITOTIC_RECOMBINATION	36			-2.890	0.000			2
GO_TELOMERE_MAINTENANCE_VIA_RECOMBINATION	30			-2.844	0.000			2
GO_DNA_DEPENDENT_DNA_REPLICATION	81			-2.753	0.000			2
GO_CENTROMERE_COMPLEX_ASSEMBLY	36			-2.696	0.000			2
GO_TRANSLATIONAL_TERMINATION	88			-2.692	0.000			2
GO_NUCLEAR_CHROMOSOME_TELOMERIC_REGION	103			-2.643	0.000			2
GO_TRNA_PROCESSING	83			-2.583	0.000			2
GO_CELLULAR_PROTEIN_COMPLEX_DISASSEMBLY	114			-2.544	0.000			2
GO_DNA_SYNTHESIS_INVOLVED_IN_DNA_REPAIR	65			-2.503	0.000			2
GO_ORGANELLAR_LARGE_RIBOSOMAL_SUBUNIT	30			-2.457	0.000			2
GO_MITOCHONDRIAL_TRANSLATION	99			-2.446	0.000			2
GO_UNFOLDED_PROTEIN_BINDING	85			-2.413	0.000			2
GO_CHROMOSOME_TELOMERIC_REGION	129			-2.367	0.001			2
GO_DNA_REPLICATION_INDEPENDENT_NUCLEOSOME_ASSEMBLY	42			-2.353	0.001			2
GO_DNA_REPLICATION_INITIATION	21			-2.352	0.001			2
GO_MACROMOLECULAR_COMPLEX_DISASSEMBLY	168			-2.323	0.001			2
GO_NUCLEAR_CHROMOSOME	438			-2.311	0.001			2
GO_DNA_REPLICATION_INDEPENDENT_NUCLEOSOME_ORGANIZATION	42			-2.297	0.001			2
GO_ORGANELLAR_RIBOSOME	69			-2.288	0.001			2
GO_TRANSLESION_SYNTHESIS	37			-2.285	0.001			2

GO_DNA_SECONDARY_STRUCTURE_BINDING	22			-2.262	0.002			2
GO_NUCLEOTIDE_EXCISION_REPAIR_DNA_GAP_FILLING	24			-2.253	0.002			2
GO_DNA_DEPENDENT_DNA_REPLICATION_MAINTENANCE_OF_FIDELITY	19			-2.219	0.002			2
GO_TRANSLATIONAL_ELONGATION	104			-2.206	0.002			2
GO_HISTONE_EXCHANGE	42			-2.190	0.003			2
GO_90S_PRERIBOSOME	17			-2.189	0.003			2
GO_DNA_BIOSYNTHETIC_PROCESS	101			-2.183	0.003			2
GO_DNA_RECOMBINATION	165			-2.162	0.003			2
GO_TRNA_METABOLIC_PROCESS	130			-2.136	0.004			2
GO_REPLICATION_FORK	54			-2.125	0.004			2
GO_POSTREPLICATION_REPAIR	49			-2.098	0.005			2
GO_ANTIMICROBIAL_HUMORAL_RESPONSE	41			-2.092	0.005			2
GO_STRAND_DISPLACEMENT	21			-2.091	0.005			2
GO_NUCLEAR_TRANSCRIBED_MRNA_CATABOLIC_PROCESS_EXONUCLEOLYTIC	28			-2.087	0.005			2
GO_DNA_STRAND_ELONGATION_INVOLVED_IN_DNA_REPLICATION	23			-2.078	0.005			2
GO_REPLISOME	27			-2.068	0.006			2
GO_CHROMATIN	374			-2.060	0.006			2
GO_TRANSCRIPTION_COUPLED_NUCLEOTIDE_EXCISION_REPAIR	71			-2.036	0.007			2
GO_ATP_DEPENDENT_CHROMATIN_REMODELING	62			-2.028	0.008			2
GO_CHROMOSOMAL_REGION	266			-2.023	0.008			2
GO_PRERIBOSOME	37			-2.023	0.008			2
GO_RNA_MODIFICATION	74			-2.014	0.008			2
GO_DNA_DAMAGE_RESPONSE_DETECTION_OF_DNA_DAMAGE	36			-2.013	0.008			2
GO_NUCLEOTIDE_EXCISION_REPAIR_DNA_INCISION	38			-2.011	0.008			2
GO_POSITIVE_REGULATION_OF_DNA_BIOSYNTHETIC_PROCESS	52			-2.000	0.009			2
GO_NUCLEAR_CHROMOSOME_SEGREGATION	178			-1.997	0.009			2
GO_REGULATION_OF_INFLAMMATORY_RESPONSE_TO_ANTIGENIC_STIMULUS	15			-1.975	0.010			2
GO_SISTER_CHROMATID_SEGREGATION	143			-1.972	0.010			2
GO_TRNA_MODIFICATION	40			-1.954	0.011			2
GO_DNA_REPLICATION	169			-1.943	0.012			2
GO_NUCLEAR_HETEROCHROMATIN	30			-1.932	0.013			2
GO_MITOTIC_SISTER_CHROMATID_SEGREGATION	73			-1.931	0.013			2
GO_DNA_DIRECTED_DNA_POLYMERASE_ACTIVITY	26			-1.923	0.014			2
GO_POSITIVE_REGULATION_OF_TELOMERE_MAINTENANCE_VIA_TELOMERE_LENGTHENING	31			-1.890	0.017			2
GO_RNA_METHYLTRANSFERASE_ACTIVITY	22			-1.886	0.017			2
GO_NUCLEOTIDE_EXCISION_REPAIR	105			-1.877	0.018			2
GO_ORGANELLAR_SMALL_RIBOSOMAL_SUBUNIT	25			-1.870	0.018			2
GO_MANNOSYLTRANSFERASE_ACTIVITY	21			-1.838	0.023			2
GO_PROTEIN_REFOLDING	20			-1.837	0.023			2
GO_NUCLEOLAR_PART	48			-1.835	0.023			2
GO_DOUBLE_STRAND_BREAK_REPAIR	125			-1.835	0.022			2
GO_METAPHASE_PLATE_CONGRESSION	38			-1.821	0.025			2
GO_ENDORIBONUCLEASE_COMPLEX	19			-1.821	0.024			2

GO_INTRAMOLECULAR_TRANSFERASE_ACTIVITY	24			-1.819	0.024			2
GO_DNA_DEPENDENT_ATPASE_ACTIVITY	70			-1.809	0.026			2
GO_MEIOTIC_CHROMOSOME_SEGREGATION	47			-1.805	0.027			2
GO_SISTER_CHROMATID_COHESION	89			-1.803	0.026			2
GO_RIBONUCLEOPROTEIN_COMPLEX_BIOGENESIS	328			-1.799	0.027			2
GO_CONDENSED_CHROMOSOME_CENTROMERIC_REGION	79			-1.794	0.028			2
GO_SPLICEOSOMAL_SNRNP_ASSEMBLY	30			-1.787	0.028			2
GO_DNA_POLYMERASE_ACTIVITY	33			-1.787	0.028			2
GO_CONDENSED_CHROMOSOME	160			-1.780	0.029			2
GO_MISMATCH_REPAIR	26			-1.778	0.029			2
GO_DNA_HELICASE_ACTIVITY	46			-1.768	0.031			2
GO_MLL1_2_COMPLEX	25			-1.752	0.034			2
GO_CHROMOSOME_SEGREGATION	212			-1.745	0.035			2
GO_GENE_SILENCING	171			-1.735	0.037			2
GO_AMIDE_BIOSYNTHETIC_PROCESS	417			-1.729	0.038			2
GO_HISTONE_BINDING	143			-1.726	0.039			2
GO_DNA_STRAND_ELONGATION	28			-1.722	0.039			2
GO_ERROR_FREE_TRANSLESION_SYNTHESIS	18			-1.718	0.040			2
GO_SMALL_SUBUNIT_PROCESSOME	21			-1.714	0.041			2
GO_RNA_CAPPING	32			-1.704	0.043			2
GO_NUCLEAR_REPLICATION_FORK	35			-1.702	0.044			2
GO_7_METHYLGUANOSINE_RNA_CAPPING	32			-1.701	0.043			2
GO_INO80_TYPE_COMPLEX	16			-1.700	0.043			2
GO_CELL_SEPARATION_AFTER_CYTOKINESIS	15			-1.691	0.045			2
GO_MANNOSYLATION	26			-1.683	0.047			2
GO_DNA_LIGATION	15			-1.676	0.049			2
GO_EUCHROMATIN	27			-1.675	0.049			2
GO_NUCLEAR_EUCHROMATIN	21			-1.672	0.049			2
GO_EMBRYONIC_SKELETAL_SYSTEM_DEVELOPMENT	114			2.365	0.000			2
GO_MAMMARY_GLAND_EPITHELIAL_CELL_DIFFERENTIATION	16			2.072	0.008			2
GO_SKELETAL_SYSTEM_MORPHOGENESIS	185			2.028	0.015			2
GO_THYROID_GLAND_DEVELOPMENT	24			2.018	0.014			2
GO_ICOSANOID_RECEPTOR_ACTIVITY	15			1.984	0.023			2
GO_EMBRYONIC_CRANIAL_SKELETON_MORPHOGENESIS	44			1.958	0.033			2
GO_ANTERIOR_POSTERIOR_PATTERN_SPECIFICATION	174			1.926	0.049			2
GO_PROTEIN_K48_LINKED_UBIQUITINATION	44					2.145	0.004	3
GO_REGULATION_OF_STEM_CELL_DIFFERENTIATION	98					2.019	0.035	3
