# Supplementary material for: Dysregulated transcriptional networks in KMT2A- and MLLT10-rearranged T-ALL
Source: Biomark Res. 2018 Aug 23;6:27. doi: 10.1186/s40364-018-0141-z (PMC6107954; doi:10.1186/s40364-018-0141-z)
Supplement: Supplementary file 6 — Table S5. GSEA for canonical pathways. (PDF 276 kb) [file 40364_2018_141_MOESM6_ESM.pdf]

Supplementaty Table S5. GSEA analyses of canonical pathways in T-ALL harboring KMT2A-R and MLLT10-R compared to Others.								
Name	SIZE	KMT2A-R vs Others (1)		MLLT10-R vs Others (2)		MLLT10-R vs KMT2A-R (3)		flag
		NES	FDR	NES	FDR	NES	FDR	
REACTOME_RNA_POL_I_PROMOTER_OPENING	47	-3.245	0.000	-3.728	0.000	-2.075	0.008	123
REACTOME_PACKAGING_OF_TELOMERE_ENDS	39	-3.162	0.000	-3.442	0.000	-1.980	0.016	123
REACTOME_MEIOTIC_RECOMBINATION	70	-3.081	0.000	-3.552	0.000	-1.996	0.016	123
KEGG_RIBOSOME	79	-3.031	0.000	-1.993	0.006			12
KEGG_SYSTEMIC_LUPUS_ERYTHEMATOSUS	107	-3.020	0.000	-3.098	0.000			12
REACTOME_DEPOSITION_OF_NEW_CENPA_CONTAINING_NUCLEOSOMES_AT_THE_CENTROMERE	50	-2.974	0.000	-3.339	0.000	-1.929	0.019	123
REACTOME_TELOMERE_MAINTENANCE	62	-2.966	0.000	-3.741	0.000	-2.455	0.001	123
REACTOME_PEPTIDE_CHAIN_ELONGATION	78	-2.954	0.000	-1.797	0.019			12
REACTOME_RNA_POL_I_TRANSCRIPTION	70	-2.950	0.000	-3.551	0.000			12
REACTOME_SRP_DEPENDENT_COTRANSLATIONAL_PROTEIN_TARGETING_TO_MEMBRANE	99	-2.878	0.000					1
REACTOME_MEIOTIC_SYNAPSIS	60	-2.857	0.000	-3.272	0.000			12
REACTOME_3_UTR_MEDIATED_TRANSLATIONAL_REGULATION	86	-2.848	0.000	-1.919	0.009			12
REACTOME_TRANSLATION	124	-2.830	0.000	-1.654	0.040			12
REACTOME_NONSENSE_MEDIATED_DECAY_ENHANCED_BY_THE_EXON_JUNCTION_COMPLEX	96	-2.827	0.000	-1.648	0.041			12
REACTOME_INFLUENZA_VIRAL_RNA_TRANSCRIPTION_AND_REPLICATION	93	-2.811	0.000	-1.904	0.010			12
REACTOME_MEIOSIS	96	-2.761	0.000	-3.260	0.000			12
REACTOME_CHROMOSOME_MAINTENANCE	100	-2.671	0.000	-3.414	0.000	-1.791	0.037	123
REACTOME_AMYLOIDS	66	-2.620	0.000	-3.127	0.000			12
REACTOME_INFLUENZA_LIFE_CYCLE	127	-2.514	0.000	-1.642	0.042			12
REACTOME_FORMATION_OF_THE_TERNARY_COMPLEX_AND_SUBSEQUENTLY_THE_43S_COMPLEX	36	-2.460	0.000	-1.979	0.006			12
REACTOME_RNA_POL_I_RNA_POL_III_AND_MITOCHONDRIAL_TRANSCRIPTION	103	-2.427	0.000	-3.195	0.000			12
REACTOME_TRANSCRIPTION	178	-2.392	0.000	-2.855	0.000			12
REACTOME_ACTIVATION_OF_THE_MRNA_UPON_BINDING_OF_THE_CAP_BINDING_COMPLEX_AND_EIFS_AND_SUBSEQUENT_BINDING_TO_43S	43	-2.363	0.000	-2.101	0.003			12
KEGG_SPLICEOSOME	96	-2.275	0.001	-2.006	0.005			12
REACTOME_METABOLISM_OF_MRNA	195	-2.128	0.003	-1.709	0.031			12
REACTOME_TRANSPORT_OF_MATURE_TRANSCRIPT_TO_CYTOPLASM	44	-2.068	0.005					1
REACTOME_PROCESSING_OF_CAPPED_INTRON_CONTAINING_PRE_MRNA	107	-2.048	0.005	-1.766	0.023			12
REACTOME_MRNA_SPLICING_MINOR_PATHWAY	37	-2.015	0.007	-2.145	0.002			12
REACTOME_TRANSPORT_OF_MATURE_MRNA_DERIVED_FROM_AN_INTRONLESS_TRANSCRIPT	33	-1.972	0.010					1
REACTOME_PD1_SIGNALING	15	-1.931	0.013					1
REACTOME_ACTIVATION_OF_NF_KAPPAB_IN_B_CELLS	59	-1.857	0.022			1.745	0.034	13
REACTOME_MRNA_PROCESSING	125	-1.848	0.023					1
REACTOME_SIGNALING_BY_CONSTITUTIVELY_ACTIVE_EGFR	17	-1.844	0.023					1

REACTOME_MRNA_SPLICING	78	-1.815	0.027	-1.693	0.033			12
REACTOME_CDT1_ASSOCIATION_WITH_THE_CDC6_ORC_ORIGIN_COMPLEX	47	-1.796	0.030	-1.654	0.040			12
KEGG_PROTEIN_EXPORT	20	-1.781	0.032					1
REACTOME_ORC1_REMOVAL_FROM_CHROMATIN	58	-1.774	0.033	-2.088	0.003			12
REACTOME_M_G1_TRANSITION	69	-1.769	0.033	-2.536	0.000			12
REACTOME_SYNTHESIS_OF_DNA	80	-1.765	0.033	-2.714	0.000			12
REACTOME_ACTIVATION_OF_THE_PRE_REPLICATIVE_COMPLEX	22	-1.762	0.033	-2.706	0.000	-1.968	0.015	123
REACTOME_HIV_LIFE_CYCLE	112	-1.739	0.038	-1.700	0.032			12
REACTOME_SCFSKP2_MEDIATED_DEGRADATION_OF_P27_P21	51	-1.738	0.038					1
REACTOME_S_PHASE	95	-1.731	0.039	-2.377	0.000			12
PID_NFKAPPAB_CANONICAL_PATHWAY	22	-1.722	0.041			1.866	0.018	13
REACTOME_DOWNSTREAM_TCR_SIGNALING	31	-1.720	0.040			1.670	0.050	13
REACTOME_P53_INDEPENDENT_G1_S_DNA_DAMAGE_CHECKPOINT	47	-1.720	0.040					1
REACTOME_CYTOSOLIC_TRNA_AMINOACYLATION	20	-1.702	0.044					1
REACTOME_CYCLIN_E_ASSOCIATED_EVENTS_DURING_G1_S_TRANSITION_	60	-1.697	0.045					1
REACTOME_CDK_MEDIATED_PHOSPHORYLATION_AND_REMOVAL_OF_CDC6	45	-1.690	0.046					1
REACTOME_SCF_BETA_TRCP_MEDIATED_DEGRADATION_OF_EMI1	47	-1.689	0.045					1
BIOCARTA_IL3_PATHWAY	15	-1.686	0.045					1
BIOCARTA_IL17_PATHWAY	16	-1.675	0.048					1
REACTOME_DNA_STRAND_ELONGATION	27			-2.924	0.000	-2.359	0.001	23
REACTOME_EXTENSION_OF_TELOMERES	23			-2.893	0.000	-2.260	0.001	23
REACTOME_DNA_REPLICATION	166			-2.702	0.000			2
KEGG_DNA_REPLICATION	34			-2.693	0.000	-1.746	0.047	23
REACTOME_LAGGING_STRAND_SYNTHESIS	17			-2.608	0.000	-2.097	0.008	23
KEGG_MISMATCH_REPAIR	22			-2.604	0.000	-1.795	0.039	23
REACTOME_ACTIVATION_OF_ATR_IN_RESPONSE_TO_REPLICATION_STRESS	27			-2.523	0.000			2
REACTOME_MITOTIC_M_M_G1_PHASES	147			-2.509	0.000			2
REACTOME_TRANSCRIPTION_COUPLED_NER_TC_NER	43			-2.465	0.000	-1.823	0.035	23
REACTOME_GLOBAL_GENOMIC_NER_GG_NER	32			-2.394	0.000	-1.856	0.029	23
REACTOME_G1_S_TRANSITION	94			-2.313	0.001			2
REACTOME_NUCLEOTIDE_EXCISION_REPAIR	48			-2.270	0.001			2
REACTOME_RESOLUTION_OF_AP_SITES_VIA_THE_MULTIPLE_NUCLEOTIDE_PATCH_REPLACEMENT_PATHWAY	16			-2.228	0.001			2
REACTOME_G2_M_CHECKPOINTS	32			-2.222	0.001			2
REACTOME_FORMATION_OF_THE_HIV1_EARLY_ELONGATION_COMPLEX	32			-2.209	0.002			2
REACTOME_CELL_CYCLE	345			-2.152	0.002			2
REACTOME_DNA_REPAIR	100			-2.132	0.002			2
REACTOME_ASSEMBLY_OF_THE_PRE_REPLICATIVE_COMPLEX	56			-2.085	0.003			2
KEGG_NUCLEOTIDE_EXCISION_REPAIR	44			-2.068	0.004			2

REACTOME_ABORTIVE_ELONGATION_OF_HIV1_TRANSCRIPT_IN_THE_ABSENCE_OF_TAT	23			-2.034	0.004			2
REACTOME_BASE_EXCISION_REPAIR	18			-1.976	0.006			2
KEGG_HOMOLOGOUS_RECOMBINATION	26			-1.914	0.010			2
REACTOME_RNA_POL_II_TRANSCRIPTION_PRE_INITIATION_AND_PROMOTER_OPENING	39			-1.895	0.011			2
REACTOME_CELL_CYCLE_MITOTIC	271			-1.877	0.012			2
REACTOME_MRNA_CAPPING	28			-1.837	0.016			2
REACTOME_CELL_CYCLE_CHECKPOINTS	100			-1.827	0.017			2
KEGG_BASE_EXCISION_REPAIR	33			-1.814	0.018			2
REACTOME_KINESINS	22			-1.813	0.018			2
PID_FANCONI_PATHWAY	42			-1.813	0.018			2
REACTOME_MITOTIC_G1_G1_S_PHASES	115			-1.785	0.021			2
PID_ATR_PATHWAY	38			-1.739	0.028			2
REACTOME_METABOLISM_OF_RNA	235			-1.721	0.031			2
REACTOME_MITOTIC_PROMETAPHASE	74			-1.720	0.030			2
REACTOME_RNA_POL_II_TRANSCRIPTION	90			-1.718	0.030			2
REACTOME_RNA_POL_II_PRE_TRANSCRIPTION_EVENTS	58			-1.708	0.031			2
REACTOME_DESTABILIZATION_OF_MRNA_BY_AUF1_HNRNP_D0	48			-1.695	0.033			2
REACTOME_E2F_MEDIATED_REGULATION_OF_DNA_REPLICATION	23			-1.690	0.033			2
REACTOME_FORMATION_OF_RNA_POL_II_ELONGATION_COMPLEX_	42			-1.686	0.033			2
REACTOME_HIV_INFECTION	189			-1.619	0.048			2
BIOCARTA_AGR_PATHWAY	35			2.003	0.015			2
PID_ERBB_NETWORK_PATHWAY	15			1.951	0.023			2
REACTOME_NUCLEAR_EVENTS_KINASE_AND_TRANSCRIPTION_FACTOR_ACTIVATION	24			1.927	0.025			2
PID_TCPTP_PATHWAY	40					1.936	0.040	3
REACTOME_SIGNALING_BY_NOTCH	92					1.933	0.029	3
REACTOME_CELL_CELL_COMMUNICATION	111					1.931	0.022	3
PID_FOXO_PATHWAY	44					1.930	0.018	3
REACTOME_SIGNALING_BY_NOTCH1	61					1.928	0.016	3
PID_AVB3_OPN_PATHWAY	31					1.920	0.015	3
PID_ERBB1_INTERNALIZATION_PATHWAY	40					1.920	0.013	3
BIOCARTA_GCR_PATHWAY	19					1.916	0.012	3
PID_CD40_PATHWAY	30					1.904	0.013	3
REACTOME_NOTCH1_INTRACELLULAR_DOMAIN_REGULATES_TRANSCRIPTION	38					1.886	0.016	3
PID_MET_PATHWAY	76					1.883	0.016	3
BIOCARTA_CD40_PATHWAY	15					1.872	0.017	3
PID_NETRIN_PATHWAY	31					1.862	0.018	3
REACTOME_DOWNREGULATION_OF_SMAD2_3_SMAD4_TRANSCRIPTIONAL_ACTIVITY	17					1.853	0.020	3
ST_INTEGRIN_SIGNALING_PATHWAY	79					1.851	0.020	3
PID_ERBB4_PATHWAY	37					1.838	0.023	3
KEGG_PHOSPHATIDYLINOSITOL_SIGNALING_SYSTEM	67					1.833	0.023	3
PID_TGFBR_PATHWAY	52					1.832	0.022	3

REACTOME_EICOSANOID_LIGAND_BINDING_RECEPTORS	15					1.824	0.023	3
PID_FAK_PATHWAY	57					1.821	0.024	3
PID_ER_NONGENOMIC_PATHWAY	41					1.817	0.024	3
BIOCARTA_MET_PATHWAY	36					1.816	0.023	3
REACTOME_THROMBOXANE_SIGNALLING_THROUGH_TP_RECEPTOR	22					1.814	0.023	3
PID_S1P_S1P2_PATHWAY	23					1.814	0.022	3
KEGG_ADHERENS_JUNCTION	73					1.814	0.021	3
REACTOME_PI_METABOLISM	35					1.808	0.022	3
REACTOME_NEPHRIN_INTERACTIONS	18					1.805	0.022	3
PID_TNF_PATHWAY	43					1.800	0.024	3
KEGG_UBIQUITIN_MEDIATED_PROTEOLYSIS	122					1.794	0.025	3
REACTOME_GOLGI_ASSOCIATED_VESICLE_BIOGENESIS	49					1.790	0.027	3
ST_ADRENERGIC	35					1.788	0.026	3
REACTOME_TRANSCRIPTIONAL_ACTIVITY_OF_SMAD2_SMAD3_SMAD4_HETEROTRIMER	34					1.787	0.026	3
REACTOME_SIGNALING_BY_TGF_BETA_RECEPTOR_COMPLEX	57					1.787	0.025	3
PID_NCADHERIN_PATHWAY	31					1.784	0.026	3
PID_NECTIN_PATHWAY	30					1.781	0.026	3
REACTOME_TRANS_GOLGI_NETWORK_VESICLE_BUDDING	56					1.780	0.025	3
PID_ERBB1_RECEPTOR_PROXIMAL_PATHWAY	35					1.777	0.026	3
BIOCARTA_PYK2_PATHWAY	26					1.774	0.026	3
KEGG_CHRONIC_MYELOID_LEUKEMIA	72					1.774	0.026	3
REACTOME_TAK1_ACTIVATES_NFKB_BY_PHOSPHORYLATION_AND_ACTIVATION_OF_IKKS_COMPLEX	18					1.766	0.028	3
REACTOME_THROMBIN_SIGNALLING_THROUGH_PROTEINASE_ACTIVATED_RECEPTORS_PARS	30					1.763	0.028	3
PID_P53_REGULATION_PATHWAY	55					1.762	0.028	3
KEGG_RENAL_CELL_CARCINOMA	69					1.746	0.035	3
BIOCARTA_NTHI_PATHWAY	23					1.745	0.034	3
BIOCARTA_TGFB_PATHWAY	18					1.744	0.034	3
REACTOME_REGULATION_OF_SIGNALING_BY_CBL	18					1.743	0.033	3
PID_AP1_PATHWAY	69					1.739	0.034	3
BIOCARTA_MTOR_PATHWAY	21					1.735	0.035	3
SIG_CD40PATHWAYMAP	33					1.733	0.035	3
REACTOME_G_ALPHA1213_SIGNALLING_EVENTS	72					1.728	0.037	3
BIOCARTA_BIOPEPTIDES_PATHWAY	41					1.728	0.037	3
PID_FGF_PATHWAY	55					1.727	0.036	3
REACTOME_TGF_BETA_RECEPTOR_SIGNALING_ACTIVATES_SMADS	23					1.719	0.039	3
PID_REELIN_PATHWAY	29					1.719	0.039	3
PID_ATF2_PATHWAY	56					1.717	0.039	3
PID_LYSOPHOSPHOLIPID_PATHWAY	62					1.714	0.040	3

REACTOME_TRAF6_MEDIATED_INDUCTION_OF_NFKB_AND_MAP_KINASES_UPON_TLR7_8_OR_9_ACTIVATION	67					1.712	0.040	3
KEGG_NOD_LIKE_RECEPTOR_SIGNALING_PATHWAY	51					1.712	0.039	3
REACTOME_SYNTHESIS_OF_PIPS_AT_THE_PLASMA_MEMBRANE	23					1.708	0.041	3
PID_PDGFRA_PATHWAY	22					1.707	0.040	3
REACTOME_CYTOCHROME_P450_ARRANGED_BY_SUBSTRATE_TYPE	49					1.704	0.041	3
REACTOME_CLASS_I_MHC_MEDIATED_ANTIGEN_PROCESSING_PRESENTATION	217					1.703	0.041	3
PID_S1P_S1P3_PATHWAY	25					1.702	0.041	3
REACTOME_INSULIN_RECEPTOR_SIGNALLING_CASCADE	78					1.692	0.045	3
KEGG_PANCREATIC_CANCER	69					1.692	0.045	3
BIOCARTA_PDGF_PATHWAY	31					1.691	0.045	3
KEGG_NON_SMALL_CELL_LUNG_CANCER	52					1.691	0.044	3
PID_TRKR_PATHWAY	59					1.691	0.044	3
KEGG_EPITHELIAL_CELL_SIGNALING_IN_HELICOBACTER_PYLORI_INFECTION	62					1.685	0.046	3
KEGG_ACUTE_MYELOID_LEUKEMIA	56					1.685	0.045	3
BIOCARTA_HER2_PATHWAY	22					1.683	0.045	3
KEGG_GLIOMA	63					1.683	0.045	3
BIOCARTA_CCR5_PATHWAY	15					1.679	0.047	3
KEGG_B_CELL_RECEPTOR_SIGNALING_PATHWAY	71					1.672	0.049	3
BIOCARTA_WNT_PATHWAY	25					1.670	0.049	3
REACTOME_PI3K_CASCADE	63					1.670	0.049	3
PID_EPHB_FWD_PATHWAY	40					1.669	0.048	3
REACTOME_OXYGEN_DEPENDENT_PROLINE_HYDROXYLATION_OF_HYPOXIA_INDUCIBLE_FACTOR_ALPHA	16					1.667	0.049	3
