# Supplementary figures and images for: Dysregulated transcriptional networks in KMT2A- and MLLT10-rearranged T-ALL
Source: Biomark Res. 2018 Aug 23;6:27. doi: 10.1186/s40364-018-0141-z (PMC6107954; doi:10.1186/s40364-018-0141-z)

**Supplementary Figure S2.** Genes that were positively enriched in *MLLT10*-R compared to *KMT2A*-R in T-ALL.

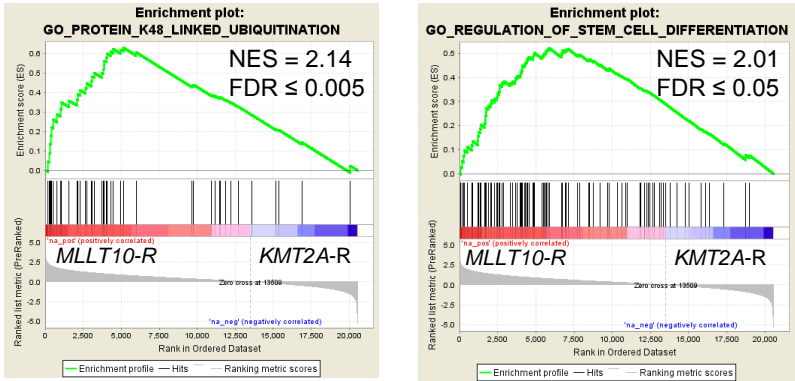

Supplement: Supplementary file 7 — Figure S2. Genes positively enriched in MLLT10. (PDF 107 kb) [file 40364_2018_141_MOESM7_ESM.pdf]
