# Supplementary material for: Dysregulated transcriptional networks in KMT2A- and MLLT10-rearranged T-ALL
Source: Biomark Res. 2018 Aug 23;6:27. doi: 10.1186/s40364-018-0141-z (PMC6107954; doi:10.1186/s40364-018-0141-z)
Supplement: Supplementary file 8 — Figure S3. KMT2A-R in B-ALL and AML. (PDF 231 kb) [file 40364_2018_141_MOESM8_ESM.pdf]

Supplementary Figure S3. *KMT2A*-R in T-ALL share similar gene expression signatures with *KMT2A*-R in AML, T-ALL or BCP-ALL.

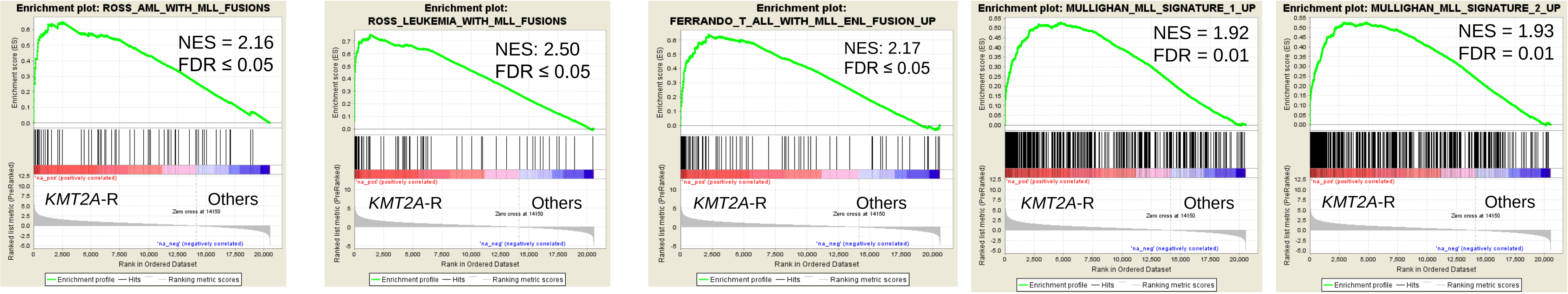

Ross *et al*., Blood, 2004

Ross *et al*., Blood, 2004

Ferrando *et al*., Blood, 2004

Mullighan *et al*., Leukemia, 2007

Mullighan *et al*., Leukemia, 2007
